# Cross-species screening platforms identify EPS-8 as a critical link for mitochondrial stress and actin stabilization

**DOI:** 10.1126/sciadv.abj6818

**Published:** 2021-10-29

**Authors:** Erica A. Moehle, Ryo Higuchi-Sanabria, C. Kimberly Tsui, Stefan Homentcovschi, Kevin M. Tharp, Hanlin Zhang, Hannah Chi, Larry Joe, Mattias de los Rios Rogers, Arushi Sahay, Naame Kelet, Camila Benitez, Raz Bar-Ziv, Gilberto Garcia, Koning Shen, Phillip A. Frankino, Robert T. Schinzel, Ophir Shalem, Andrew Dillin

**Affiliations:** 1 Department of Molecular and Cellular Biology, Howard Hughes Medical Institute, The University of California, Berkeley, Berkeley, CA 94720, USA.; 2 Leonard Davis School of Gerontology, University of Southern California, Los Angeles, CA 90089.; 3 Center for Bioengineering and Tissue Regeneration, Department of Surgery, University of California, San Francisco, San Francisco, CA 94143, USA.; 4 Department of Genetics, Perelman School of Medicine, University of Pennsylvania, Philadephia, PA 191004, USA.

## Abstract

The dysfunction of mitochondria is associated with the physiological consequences of aging and many age-related diseases. Therefore, critical quality control mechanisms exist to protect mitochondrial functions, including the unfolded protein response of the mitochondria (UPR^MT^). However, it is still unclear how UPR^MT^ is regulated in mammals with mechanistic discrepancies between previous studies. Here, we reasoned that a study of conserved mechanisms could provide a uniquely powerful way to reveal previously uncharacterized components of the mammalian UPR^MT^. We performed cross-species comparison of genetic requirements for survival under—and in response to—mitochondrial stress between karyotypically normal human stem cells and the nematode *Caenorhabditis elegans*. We identified a role for EPS-8/EPS8 (epidermal growth factor receptor pathway substrate 8), a signaling protein adaptor, in general mitochondrial homeostasis and UPR^MT^ regulation through integrin-mediated remodeling of the actin cytoskeleton. This study also highlights the use of cross-species comparisons in genetic screens to interrogate cellular pathways.

## Teaser

Cross-species genetic screens for mitochondrial regulators revealed EPS-8 as a link between actin stability and mitochondria.

## INTRODUCTION

Mitochondrial fitness and function are inarguably important to cell viability, due to their numerous functions, including energy production, apoptotic and necrotic cell death regulation, calcium and amino acids storage, lipid oxidation, and heat production. Mitochondrial dysfunction is linked to aging and several age-related diseases, including Alzheimer’s disease, Parkinson’s disease, and metabolic syndrome (*1*). This means that proper functioning of mitochondria is essential for a healthy physiological state. However, almost counterintuitively, a growing number of studies have shown that perturbations to mitochondrial function can actually lead to life-span extension (*2*–*5*). The primary reason for these seemingly contradictory observations is that impairment of mitochondria triggers the unfolded protein response of the mitochondria (UPR^MT^), which results in beneficial effects on organismal health (*6*).

UPR^MT^ is most well characterized in *Caenorhabditis elegans*, where the transcriptional regulator ATFS-1 (activating trancription factor associaited with stress 1) serves as a signal between the mitochondria and the nucleus. ATFS-1 contains both a nuclear localization sequence and a mitochondrial signal sequence and is preferentially imported into the mitochondria where it is subsequently degraded by mitochondrial Lon proteases. When mitochondrial import is compromised, such as under conditions of stress, ATFS-1 import is reduced and can accumulate in the nucleus to activate UPR^MT^ (*7*). Several other transcriptional regulators work either in concert with, or independent of, ATFS-1 to regulate UPR^MT^, including the transcription factor DVE-1 and its ubiquitin-like cofactor UBL-5 (*8*, *9*). Nuclear localization and transcriptional activation by DVE-1 are further promoted by the histone methyltransferase MET-2 and its cofactor LIN-65, which also induce epigenetic changes required for transmission of mitochondrial stress signaling between cells and through generations (*10*). Last, histone demethylases JMJD-1.2 (jumonji transcription factor domain protein 1.2) and JMJD-3.1 remodel chromatin to facilitate access to promoters of numerous UPR^MT^ target genes (*11*).

The mechanisms underlying UPR^MT^ are poorly understood in human cells, and whether there exists a true UPR^MT^ pathway itself is controversial. One study proposed the role of ATF5 (activating transcription factor 5) as a critical transcription factor that mediates mammalian UPR^MT^, such that it can be localized to mitochondria, promoted mitochondrial function and genes involved in repairing mitochondria, and rescued the loss of ATFS-1 in *C. elegans* (*12*). However, a large-scale screening study showed that the primary response to mitochondrial dysfunction caused by drugs targeting mitochondrial translation, respiration, and import was the integrated stress response (ISR) mediated by ATF4 (*13*). It is possible that the lack of understanding can be due to the inherent nature of an in vitro cell culture system, as growth on stiff surfaces caused major changes to mitochondrial metabolism (*14*). Overall, it is still unclear how UPR^MT^ is regulated in human cells, and it is possible that the discrepancy between studies is due to the many differences that exist between cell types and their optimal culturing conditions.

Here, we reasoned that an evolutionary conservation perspective could provide a uniquely powerful way to reveal previously uncharacterized yet important components of how the mitochondrion responds to insults to its proteome. Specifically, we combined the best experimental features of two unrelated experimental systems to understand the transcriptional response to—and genes required for—surviving mitochondrial stress. First, we used the power of CRISPR-Cas9–driven reverse genetics and the tools of transcriptome analysis to identify a list of candidate human genes that, in a specific experimental setting, appear connected to response to mitochondrial stress at the functional or transcription regulation level. We elected to do these experiments in karyotypically normal neural progenitor cells (NPCs) under physiological levels of oxygen (4%, v/v) because they are proliferative and represent a physiologically relevant cell type for understanding mitochondrial function.

We then performed an evolution-based filtering of candidate “hits” obtained from these human cell culture experiments into a well-studied multicellular eukaryote, *C. elegans*. The in vivo model system allows for functional studies of mitochondrial stress response without the potential pitfalls that exist under standard culturing conditions. For example, artifacts of mitochondrial form and function have been seen in high glucose, differences in oxygen levels, or the high mechanical tension that comes from growth on plastic petri dishes (*14*, *15*). Thus, cross-examination across two distinct systems, each with their unique strengths, would create a robust and highly confident candidate gene list important for mediating mitochondrial quality control. Through these studies, we identified a previously unidentified link between mitochondria and actin homeostasis through EPS-8 (epidermal growth factor receptor pathway substrate 8), which exhibits a seemingly contrasting function in promoting actin health at the cost of mitochondrial function.

## RESULTS

In our work to identify cellular components of the mammalian UPR^MT^, we chose to rely on an experimentally robust mitochondrial stress: the chemical down-regulation of electron transport chain (ETC) function. Using rotenone or antimycin A to induce mitochondrial stress, we performed CRISPR-Cas9 screening and transcriptome analysis to identify critical genes involved in responding to mitochondrial stress. Screens were carried out using nontransformed, karyotypically normal NPCs. NPCs are self-renewing cells that can give rise to multiple neuronal and glial cell types. Mitochondrial dynamics have been shown to regulate NPC self-renewal and differentiation (*16*, *17*), highlighting the importance of understanding mitochondrial stress regulation in NPCs. In addition, neurons have been implicated in multiple facets of mitochondrial stress response, whereby they serve as sentinel sensors of mitochondrial stress to coordinate an organism-wide response (*6*, *11*, *18*, *19*). Last, as nontransformed, karyotypically normal cells, they are not commonly used in CRISPR screens, allowing a vastly different paradigm for genetic screening in comparison to previously published datasets using karyotypically abnormal cancer cell lines.

**Fig. 1.**
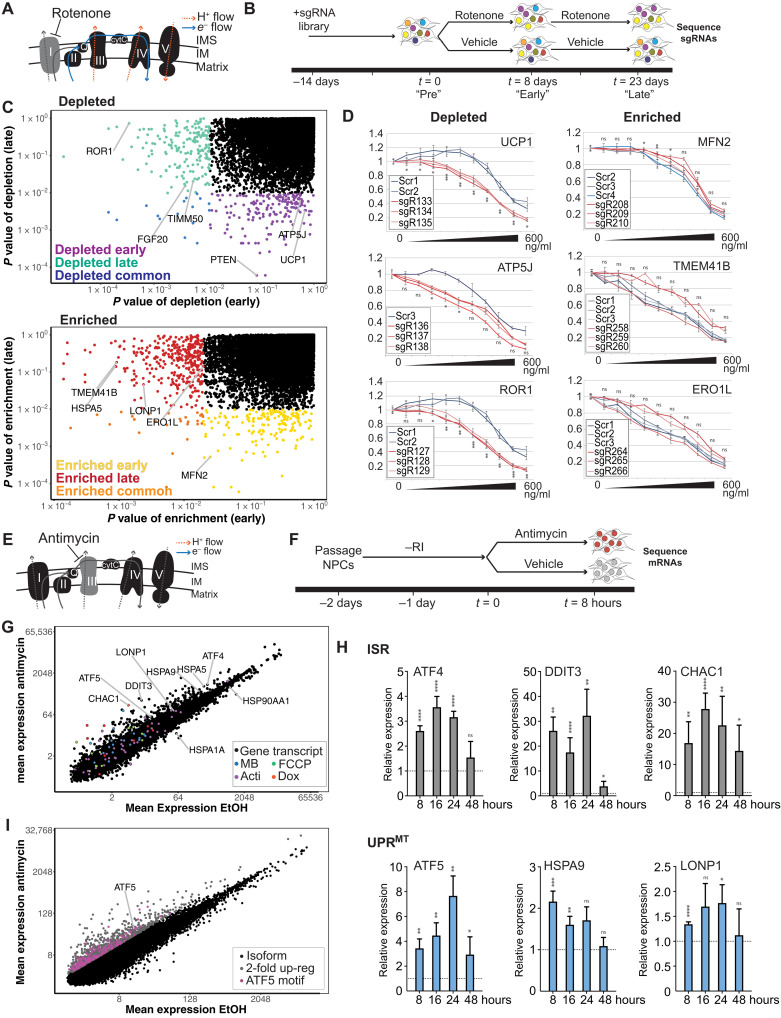
Cas9 screening and transcriptome analysis to identify critical genes for response to mitochondrial stress. (**A**) Model of the effect of rotenone on electron flow through ETC. IM, inner membrane; IMS, inner membrane space; cytC, cytochrome C. (**B**) Time course of rotenone (3.5 ng/ml) screen: pretreatment (Pre), 8 days (Early), and 23 days (Late). (**C**) Unadjusted *P* values associated with depletion (top) or enrichment (bottom) of individual genes. For each graph, *x* axis shows *P* value at 8 days (early) and *y* axis shows *P* value at 23 days (late). Colored points indicate genes selected for follow-up in *C. elegans*. (**D**) Rotenone dose-response survival of cell pools enriched for gene knockouts versus negative controls. *X* axis: Rotenone concentration in nanograms per milliliter. *Y* axis: Relative luminescence versus control. ns, not significant. *P < 0.05, **P < 0.01, and ***P < 0.001 using *t* test versus control. (**E**) Model of the effect of antimycin on electron flow through ETC. (**F**) Time course of antimycin (10 μg/ml) transcriptome experiment. Rock inhibitor (“RI”) was removed the day following passaging. (**G**) Mean expression of individual isoforms [in transcripts per million (TPM)] is plotted. All transcripts up-regulated twofold in (*13*) are indicated in the specified colors. MB, MitoBLOCK; Acti, actinonin; Dox, doxycycline. (**H**) Genes from the indicated stress responses [ISR and UPR^MT^ were analyzed by reverse transcription quantitative polymerase chain reaction (RT-qPCR) over the indicated timeline]. Mapped are fold change for two to six biological replicates of antimycin treatment to ethanol (EtOH) treatment with SEM and paired *t* test. *P < 0.05, **P < 0.01, ***P < 0.001, and ****P < 0.0001. (**I**) Individual isoforms are plotted. Raw data are found in table S2. All isoforms with twofold up-regulation (gray) were filtered for those with an ATF5 binding motif in the promoter (pink).

We performed a genome-wide CRISPR screen for genes involved in NPC response to low-dose rotenone, a known complex I inhibitor (Fig. 1, A and B, and see Materials and Methods for details). This treatment induced slow growth (fig. S1A) without causing notable cell death. Using single-guide RNA (sgRNA) abundance as a proxy for the effect of the loss of the target gene(s) for cell survival and proliferation, we first analyzed depletion of sgRNAs before stress application (Fig. 1B, “Pre”) and observed that genes associated with essential processes were significantly depleted (table S1). Smaller effect sizes were observed for a distinct panel of genes following 8 and 23 days of rotenone treatment [Fig. 1, B (“Early” and “Late”) and C (top)], when compared to time points following treatment with vehicle. Specifically, using a *P* value cutoff of <0.01, 173 genes were depleted in the early time point, 206 genes were depleted in the late time point, and 15 genes were common between both datasets. These genes include those encoding proteins that function in the mitochondrion (e.g., the importer TIMM50, translocase of inner mitochondrial membrane 50), the mitochondria membrane uncoupling protein UCP1, adenosine triphosphate (ATP) synthase ATP5J, those known to be required for survival under mitochondrial stress [phosphatase and tensin homolog (*20*)], and those connected to neurodegeneration [e.g., fibroblast growth factor 20, which is implicated in sporadic Parkinson’s disease (*21*), symptoms of which rotenone induces in rodents (*22*)]. We also identified ROR1 (receptor tyrosine kinase like orphan receptor 1), a gene with no clear connection to mitochondria. Further, using a *P* value cutoff of <0.01, sgRNAs targeting 201 genes were enriched in cells at the early time point and 198 were enriched late, with 10 genes in common. These genes encode several proteins important for mitochondrial proteostasis, including the mitochondrial fusion regulator MFN2 (mitofusin 2), the deacetylase SIRT3 (sirtuin 3), mitochondrial protease LONP1 (lon peptidase 1), and TMEM41B (transmembrane protein 41B), which was recently shown to regulate mitophagy (*23*). Loss of ERO1L, an endoplasmic reticulum (ER)–localized oxidoreductase, and ER chaperone HSPA5 (heat shock protein family A member 5) improved survival (Fig. 1C). Follow-up gene-by-gene knockout analysis under rotenone stress broadly agreed with data from the pooled screen (Fig. 1D and fig. S1, A and B). We were unable to assess the effects of these genes on growth in stronger ETC inhibitors, such as antimycin.

To identify genes that may act in an acute fashion in response to stress (identification of which can be challenging under conditions of cell expansion in a CRISPR-based screen), we sought to characterize the transcriptomic response to ETC stress. To increase sensitivity of this analysis, we used a different inhibitor of the ETC, antimycin, which exerts its effects on complex III, and whose effect more completely abrogates ETC function (Fig. 1E). We performed RNA sequencing (RNA-seq) following an 8-hour treatment (Fig. 1F). Antimycin treatment significantly up-regulated or down-regulated expression of 202 and 67 genes twofold, respectively (table S2). We found an overlap in the gene list relative to that from a cognate study in HeLa cells (*13*), particularly for many of our most up-regulated genes (Fig. 1G). Overall, the similarity was modest, suggesting important differences between primary and transformed cells or the exact nature of the mitochondrial stress applied. Reverse transcription quantitative polymerase chain reaction (RT-qPCR) analysis of effects on individual genes (Fig. 1H) confirmed modest up-regulation of (i) the master ISR regulator ATF4 and robust up-regulation of its targets [CHAC1 (ChaC glutathione specific gamma-glutamylcyclotransferase 1) and DDIT3 (DNA damage inducible transcript 3)], (ii) the UPR^MT^ regulator ATF5 and modest up-regulation of effectors of the UPR^MT^ (HSPA9 and LONP1) (Fig. 1H), and (iii) the ER unfolded protein response (UPR^ER^) chaperone HSPA5 (fig. S1D). Expression of key heat shock proteins, HSPA1A and HSP90AA1, was markedly reduced (fig. S1D). These data show that human NPCs respond to acute ETC inhibition by broadly modulating the expression of genes involved in stress response, only a fraction of which represents known components of the mitochondrial stress response.

Considering the minimal overlap between ours and previously published datasets beyond the ISR (*13*), we next sought to computationally extract candidate genes more likely to participate in a canonical UPR^MT^ for cross-species analysis. Given the reported role of the transcription factor ATF5 in UPR^MT^, we sought to understand the impact of ATF5-driven UPR^MT^ in the antimycin response (*12*). To do this, we identified all transcript isoforms whose expression increased twofold (Fig. 1I, gray) and computationally analyzed the promoters for the presence of matches to the position weight matrix (PWM)–represented consensus binding site for ATF5 (see Materials and Methods for details). Of the 6163 isoforms representing 3832 genes, 430 contained one or more such matches (Fig. 1I, pink); these genes were prioritized for further analysis.

**Fig. 2.**
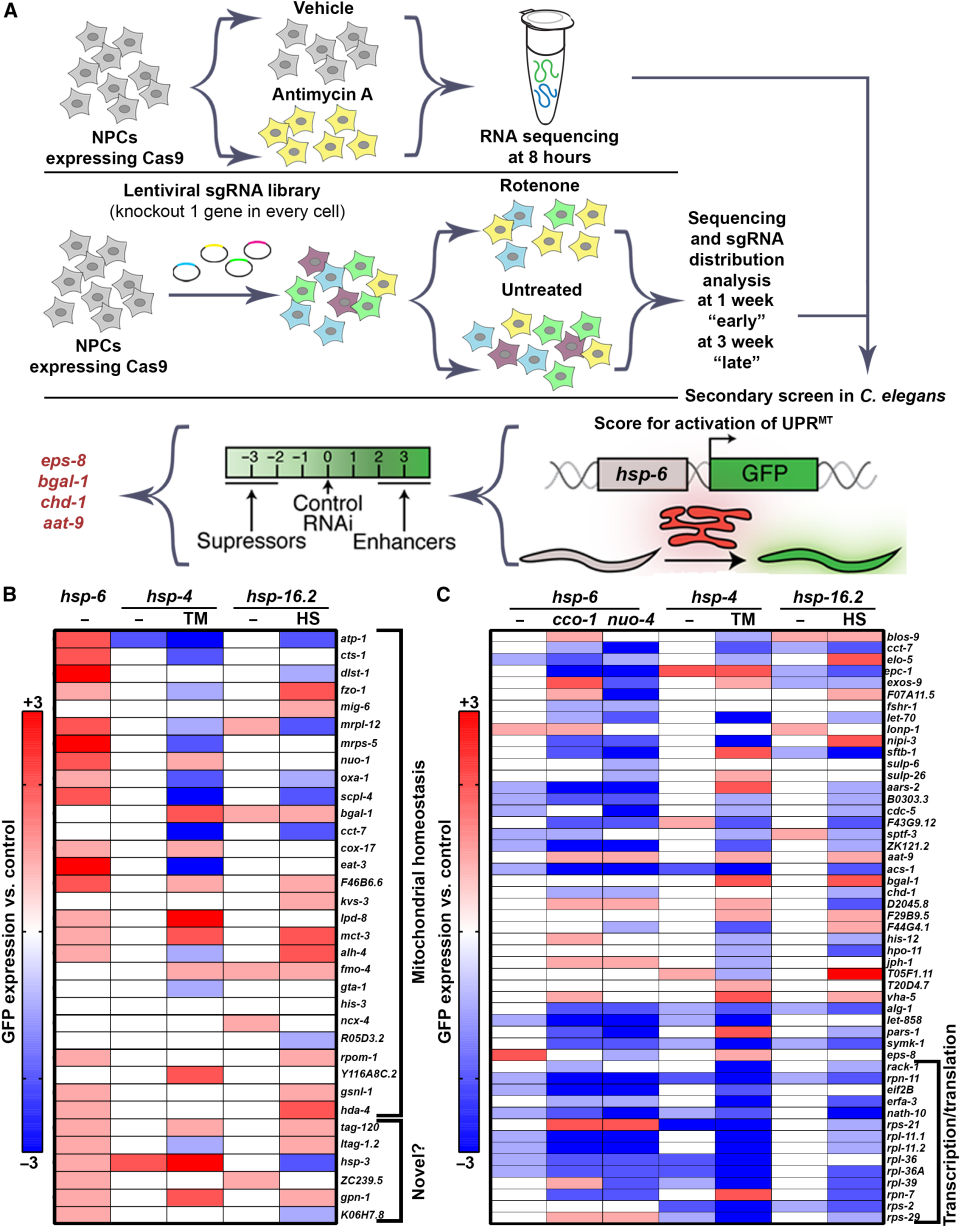
Secondary screening in *C. elegans* to identify regulators of cellular stress response. (**A**) Schematic of cross-species screening. CRISPR-Cas9 screening and transcriptome analysis were performed in NPCs, and top hits were screened in *C. elegans* using the UPR^MT^ reporter, *hsp-6p::GFP*. (**B**) The top “basal inducers” described as genes that, when knocked down alone, induce UPR^MT^ were rescreened and imaged for effects on *hsp-6p::GFP* (UPR^MT^), *hsp-4p::GFP* (UPR^ER^), and *hsp-16.2p::GFP* (HSR) induction. Increased or decreased expression of UPR^MT^, UPR^ER^, or HSR are scored on an integer scale from −3 to +3 compared to controls. For basal induction, animals grown on empty vector control and left untreated serve as a 0 level control. For stress induction, animals grown on empty vector control and treated with specified stress (TM, tunicamycin; HS, heat shock) serve as a 0 level control. Blue indicates decreased expression, and red indicates increased expression. All numerical data can be found in table S5, and all images can be found in the Supplementary Materials. (**C**) The top “suppressors” described as genes that when knocked down decreased induction of UPR^MT^ under stress via either *cco-*1 or *nuo-*4 knockdown were rescreened and imaged for effects on *hsp-6p::GFP* (UPR^MT^), *hsp-4p::GFP* (UPR^ER^), and *hsp-16.2p::GFP* (HSR) induction. Increased or decreased expression of UPR^MT^, UPR^ER^, or HSR are scored on an integer scale from −3 to +3 compared to controls. For basal induction, animals grown on empty vector control and left untreated serve as a 0 level control. For stress induction, animals grown on empty vector control and treated with specified stress (20% *cco-1* or *nuo-4* RNAi) serve as a 0 level control. Blue indicates decreased expression and red indicates increased expression. All numerical data can be found in table S6, and all images can be found in the Supplementary Materials.

Having performed the above reverse genetic, transcriptomic, and computational analysis of NPC response to ETC inhibition, we merged the lists of genes identified by the three approaches to yielding ~1000 human gene candidates for cross-species analysis. Next, we computationally extracted those with robustly identifiable orthologs in the *C. elegans* genome (*24*), which yielded a list of ~600 genes (table S3). To identify regulators of UPR^MT^, we performed secondary screening of our top candidate genes using a robust and reliable reporter to measure UPR^MT^ activity (*25*). Here, we use a transcriptional reporter for UPR^MT^ where green fluorescent protein (GFP) expression is driven under the promoter of a mitochondrial chaperone, HSP-6, orthologous to mammalian HSPA9 (*hsp-6p::GFP*) (*26*). The level of GFP serves as a qualitative and semiquantitative measure of UPR^MT^ induction. We performed an RNA interference (RNAi)–knockdown screen to identify regulators of basal or stress-induced UPR^MT^ activation. Specifically, we systematically knocked down each of the ~600 genes originating from the human cell culture experiments to identify one of the following: (i) genes that induce UPR^MT^ when knocked down or (ii) genes that suppress stress-induced UPR^MT^ activation, where stress is applied by suppressing ETC function by RNAi knockdown of either *cco-1/cox-5b*, the complex IV cytochrome c oxidase subunit Vb/COX4, or *nuo-4*, the complex I reduced form of nicotinamide adenine dinucleotide dehydrogenase protein (Fig. 2A and details of screen in Materials and Methods) (*6*, *27*).

As observed in our raw data available in table S4, most of our phenotypes are robustly reproducible across two biological replicates. To increase the strength and reliability of our dataset, we performed a third replicate of our top candidates, including 34 genes that induced UPR^MT^ when knocked down and 51 genes that suppressed UPR^MT^ induction under stress via RNAi knockdown of *cco-1*, *nuo-4*, or both (Fig. 2, B and C, and tables S5 and S6). Moreover, to eliminate subjective bias, we provide raw fluorescent images of each of our top hits in the Supplementary Materials to supplement the semiquantitative data we provide.

While several genes failed to show identical results in our final replicate, these were a small fraction of the genes in our dataset, but they highlight critical importance of multiple layers of validation as we have performed here. Most genes that induce UPR^MT^ induction upon RNAi knockdown are known components of mitochondria, including *fzo-1* (MFN2) (*28*), *mrps-5* (MRPS5, mitochondrial ribosomal protein S5) (*29*), *atp-1* (ATP5A1) (*30*), and *nuo-1* (NDUFV1, NADH:Ubiquinone oxidoreductase core subunit V1) (*30*) (Fig. 2B). The identification of genes previously implicated in UPR^MT^ gives us very high confidence in our datasets. The most notable inducers of UPR^MT^ not previously implicated in mitochondrial health include *gpn-1* (glypican 6), a heparan-sulfate proteoglycan (*31*); *ltah-1.2* (LTA4H, leukotriene A4 hydrolase), a regulator of inflammatory signals through leukotriene synthesis (*32*); *hsp-3* (HSPA5), a chaperone primarily implicated in regulating ER homeostasis (*33*), which, importantly, was also identified in the NPC screens where its knockout improved growth in rotenone and its expression is up-regulated in antimycin; *ZC239.5* (TNFAIP1, TNF alpha induced protein 1), a substrate-specific adaptor for an E3 ligase that mediates the ubiquitination of RhoA (Ras homolog family member A) (*34*); and *K06H7.8* (tau-tubulin kinase 2), a tau-tubulin kinase important for ciliogenesis (*35*).

To determine the impact of our identified genes in overall stress response beyond the UPR^MT^, we performed similar imaging of canonical transcriptional reporters of other stress responses, including the UPR^ER^ and heat-shock response (HSR). Specifically, we measured *hsp-4p::GFP* (HSPA5/BiP) induction in the presence and absence of tunicamycin, which causes ER stress through inhibition of N-linked glycosylation and robustly induces the activation of UPR^ER^ (*25*, *36*, *37*), and *hsp-16.2::GFP* (CRYAB, crystallin alpha B) in the presence and absence of heat stress to measure the activation of HSR (Fig. 2, B and C) (*38*). Consistent with previous findings (*39*), we find that knockdown of a select few genes that induce UPR^MT^ and therefore likely cause mitochondrial stress also affects the cytosolic HSR. It is likely that under conditions of mitochondrial stress, HSF-1 (heat-shock factor 1) can be mobilized to affect targets of the cytosolic HSR to improve organismal homeostasis (*14*, *39*, *40*). RNAi knockdown of most of our candidate genes failed to induce the HSR, and all failed to induce the UPR^ER^, suggesting that our screening paradigm was highly specific in identifying genes involved in mitochondrial homeostasis. However, many genes that induced UPR^MT^ altered induction of both UPR^ER^ and HSR under stress. While knockdown of many genes enhanced the cell’s capacity to respond to heat stress, again providing further evidence that mitochondrial dysfunction mobilizes HSF-1, the effect on UPR^ER^ were split evenly between suppressors and enhancers, suggesting complex intercommunication between the mitochondria and ER under stress. For the most part, severe mitochondrial dysfunction (i.e., higher induction of UPR^MT^) was correlated with decreased UPR^ER^ and HSR induction under stress, inviting the intriguing hypothesis that hyperactivation of a single stress response may titrate away the cell’s capacity to induce other stress responses.

Overall, suppressors of stress-induced UPR^MT^ displayed much more consistent results across replicates and had much larger—and likely more significant—changes compared to inducers of UPR^MT^ (Fig. 2C and table S6). Therefore, we curated a larger list of these suppressor genes for further analysis. Initial characterization of our list of UPR^MT^ suppressors identified many genes encoding proteins involved in ribosomal processing and function, which are expected to decrease global translation (Fig. 2C). RNAi knockdown of these genes also generally suppressed the activation of other stress responses, including the UPR^ER^ and cytosolic HSR, providing further evidence that our screening paradigm was robust and capable of identifying genes involved in regulating all three stress responses. Beyond ribosomal genes, we found several other genes that differentially affected multiple stress responses, including *blos-9* (BORCS8, BLOC-1 related complex subunit 8), which regulates axonal transport of synaptic vesicles and lysosomes (*41*); *elo-5* (ELOVL6, ELOVL fatty acid elongase 6), involved in monomethyl branched-chain fatty acid synthesis (*42*); *epc-1* (EPC2, enhancer of polycomb homolog 2), a conserved histone acetyltransferase involved in acetylation of histones H4 and H2A (*43*); *exos-9* (EXOSC9, exosome component 9), an essential component of the RNA exosome complex (*44*); *sftb-1* (SF3B1, splicing factor 3b subunit 1), part of a pre-mRNA splicing complex (*45*); and *nipi-3* (TRIB3, tribbles pseudokinase 3) involved in the ISR (*46*).

In an effort to prioritize our large list of candidate genes, we ranked all candidate genes for the following conditions: (i) robust and reproducible induction of basal *hsp-6p::GFP* upon knockdown, suggesting that this gene is required for mitochondrial homeostasis and its knockdown induces mitochondrial stress; (ii) suppression of either *cco-1* or *nuo-4*–induced UPR^MT^, suggesting that the gene is required for the induction of UPR^MT^; and (iii) little to no effect on the UPR^ER^ or HSR, suggesting that it was a specific regulator of mitochondrial homeostasis rather than a global regulator of cellular health. Using these criteria, *eps-8* (EPS8/EPS8L in mammals) ranked the highest, as a robust and reproducible inducer of UPR^MT^ when knocked down, a suppressor of stress-induced UPR^MT^, and with minimal effect on the UPR^ER^ and HSR.

**Fig. 3.**
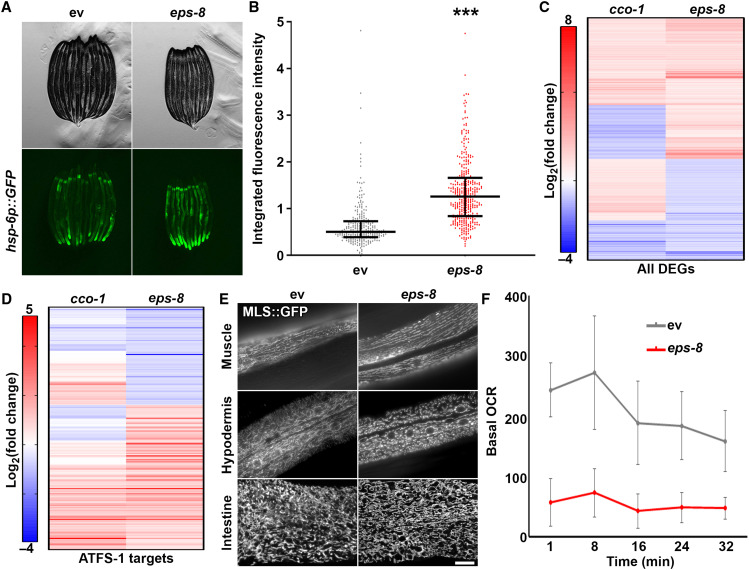
Loss of *eps-8* results in defects in mitochondrial homeostasis. (**A**) Contrast-matched fluorescent micrographs of day 1 adult *hsp-6p::GFP* animals grown on empty vector control (ev) or *eps-8* RNAi from hatch. (**B**) Quantification of *hsp-6p::GFP* in day 1 adult animals grown on empty vector control (gray) or *eps-8* RNAi (red) from hatch. Lines represent median and interquartile range, with each dot representing a single animal. *n* = 257 for empty vector control and *n* = 358 for *eps-8*. Data are representative of three independent trials. ***P < 0.001 using nonparametric Mann-Whitney testing. (**C**) RNA-seq of animals grown on empty vector control or *eps-8* RNAi. Heatmap indicates log_2_(fold change) of genes where red indicates up-regulated and blue indicates down-regulated genes. Here, canonical UPR^MT^ genes are represented as those that were differentially expressed by RNAi knockdown of *cco-1*. See table S7 for actual values of log_2_(fold change). (**D**) Differentially expressed genes (DEGs) from (C) that were previously identified as bonafide ATFS-1 targets (*47*) were extracted and plotted separately. (**E**) Representative fluorescent images of day 1 adult animals expressing a mitochondria-targeted GFP in *myo-3p* (muscle), *col-19p* (hypodermis), and *gly-19p* (intestine). Scale bar, 10 μm. (**F**) Basal oxygen consumption rate (OCR) was measured in N2 animals grown on empty vector control or *eps-8* RNAi using the Seahorse XFe96 Analyzer. Data are represented as means ± SD measured across the 10 wells and is representative of three independent trials.

First, to further validate that *eps-8* is a true regulator of UPR^MT^, we quantified UPR^MT^ induction across a large population of worms using a large particle biosorter (*25*). We confirmed across multiple biological replicates of several hundred worms that knockdown of *eps-8* resulted in a significant increase in UPR^MT^ induction (Fig. 3, A and B). Furthermore, this induction of UPR^MT^ was completely suppressed by loss of *atfs-1*, the gene encoding one of the primary transcriptional regulators of UPR^MT^ induction (fig. S2, A and B) (*7*). To further corroborate the effects of *eps-8* knockdown on UPR^MT^ induction, we tested the effect of *eps-8* knockdown on nuclear localization of DVE-1, another important transcriptional regulator of UPR^MT^, which has previously been validated to be a reliable indicator for UPR^MT^ activity (*10*). We find that similar to the induction of *hsp-6p::GFP*, RNAi knockdown of *eps-8* resulted in a dramatic increase in nuclear-localization of DVE-1::GFP (fig. S2C). Last, we performed transcriptome analysis to measure global changes in transcription and find that knockdown of *eps-8* shares many changes in gene expression to induction of UPR^MT^ via *cco-1* knockdown (Fig. 3C and table S7) (*11*). While the changes are not fully overlapping because of the pleiotropic effects of *eps-8*, the similarities are even more apparent when comparing predicted targets of ATFS-1 in these two datasets (Fig. 3D) (*47*). The transcriptome of *eps-8* knockdown shows little to no overlap when compared to induction of another stress response, the UPR^ER^ through *xbp-1s* overexpression in neurons (*48*), suggesting that loss of *eps-8* does not result in general activation of stress (fig. S2, C and D, and table S8). Together, these data provide further evidence that *eps-8* expression directly influences UPR^MT^.

To our surprise, loss of *eps-8* selectively suppressed induction of UPR^MT^ that occurs with knockdown of *nuo-4*, but not of *cco-1* (fig. S3, A and B). It is possible that the differences between these two stressors are the location of ETC inhibition. NUO-4 (NADH ubiquinone oxidoreductase) is a complex I protein, while CCO-1 (cytochrome c oxidase-1) is part of complex IV, and it has been previously described that complex I is one of the primary producers of reactive oxygen species (*49*) along with complex III (*50*, *51*). We find that RNAi knockdown of *nuo-4*, but not *cco-1*, results in activation of oxidative stress response (OxSR) using the reporter for SKN-1 (skinhead-1)/NRF2 (nuclear factor, erythroid 2 like 2) activity, *gst-4p::GFP* (*52*). Moreover, loss of *eps-8* partially suppressed the activation of OxSR upon knockdown of *nuo-4*, suggesting that EPS-8 may also alter OxSR, potentially through its effects of mitochondrial homeostasis (fig. S3, C and D).

We tested whether the cause of UPR^MT^ induction upon loss of *eps-8* was due to a functional role of EPS-8 in regulating mitochondrial homeostasis in general. We analyzed mitochondrial morphology using a mitochondria-targeted GFP expressed specifically in the muscle, hypodermis, or intestine of *C. elegans*. We find that loss of *eps-8* resulted in fragmentation of mitochondria in the muscle and hypodermis (Fig. 3E), which is likely linked to severe defects in oxygen consumption rate (Fig. 3F). Loss of *eps-8* had no effect on mitochondrial morphology in intestinal cells, suggesting differences in requirement of EPS-8 in specific cell types (Fig. 3E). This is consistent with data from a parallel study in this issue that identified *eps-8* in a screen for previously uncharacterized regulators of aging through protein homeostasis (*53*). Koyuncu *et al.* (*53*) identified EPS-8 as a target for ubiquitin-mediated degradation during aging, such that decreased function of the ubiquitin-proteasome system with age results in increased levels of EPS-8 due to failure of turnover. In this study, Koyuncu *et al.* (*53*) similarly found tissue-specific effects of *eps-8* knockdown, such that reversing the age-associated accumulation of EPS-8 in the muscle and neurons via RNAi knockdown of *eps-8* resulted in increased life span, while knockdown in the intestine had no effect (*53*).

To determine the mechanism whereby EPS-8 modulates mitochondrial homeostasis, we focused on two previously identified mechanisms of EPS-8. First, EPS-8 has been shown to promote the activity of RAC through SOS-1 by catalyzing the exchange of guanosine diphosphate for guanosine 5′-triphosphate on RAC protein (*54*, *55*). Koyuncu *et al.* (*53*) showed that EPS-8–mediated effects on RAC directly affected life span, such that RNAi knockdown of RAC components *mig-2* and *rac-2* phenocopied *eps-8* knockdown to promote life span. Moreover, the life-span defects of mutants with increased EPS-8 function were ameliorated by knockdown of *mig-2* (*53*). In stark contrast to these phenotypes, we find that knockdown of *ced-10*, *mig-2*, and *rac-2* failed to phenocopy *eps-8* knockdown in mitochondrial homeostasis. Specifically, knockdown of *ced-10*, *mig-2*, and *rac-2* failed to induce UPR^MT^ or alter mitochondrial morphology (fig. S4). These data suggest that unlike the findings by Koyuncu *et al.* (*53*), our model of EPS-8–mediated effects on mitochondrial homeostasis is independent of RAC.

**Fig. 4.**
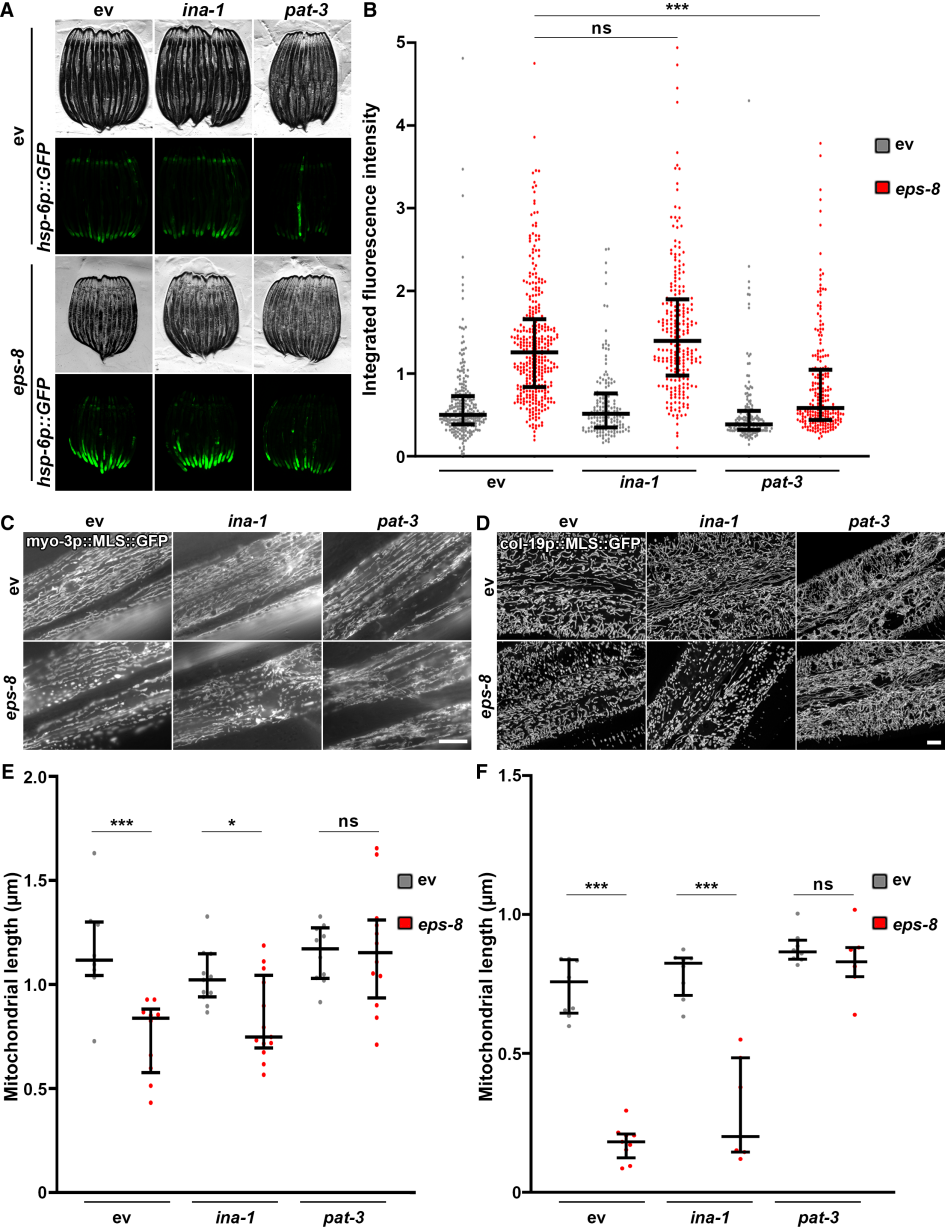
EPS-8 mediates mitochondrial homeostasis through integrin. (**A**) Fluorescent micrographs of day 1 adult *hsp-6p::GFP* animals grown on ev control or *eps-8* RNAi mixed in a 1:1 ratio with empty vector control, *ina-1*, or *pat-3* RNAi from hatch. All images are contrast-matched. (**B**) Quantification of *hsp-6p::GFP* in day 1 adult animals grown on empty vector control (gray) or *eps-8* RNAi (red) mixed in a 1:1 ratio with empty vector control, *ina-1*, or *pat-3* RNAi from hatch. Lines represent median and interquartile range, with each dot representing a single animal. *n* = 156 to 358 per strain. Data are representative of three independent trials. ***P < 0.001; ns, *P* > 0.10 using nonparametric Mann-Whitney testing. (**C** and **D**) Representative fluorescent images of day 1 adult animals expressing a mitochondria-targeted GFP from a muscle-specific promoter, *myo-3p* (C) or a hypodermal-specific promoter, *col-19p* (D). Animals were grown on empty vector control or *eps-8* RNAi mixed in a 1:1 ratio with empty vector control, *ina-1*, or *pat-3* RNAi from hatch and imaged directly on glass slides as described in Materials and Methods. Muscle images were captured on a standard wide-field Zeiss AxioObserver Z1. Hypodermal images were captured on an LSM900 Airyscan microscope. Scale bars, 10 μm. (**E** and **F**) Quantification of (C) and (D) using MitoMAPR. For muscle (E), quantification was performed on two to three muscle cells for 6 to 10 animals per sample on single slice images. For hypodermis (F), quantification was performed on single image max projections for 6 to 10 animals per sample. *P < 0.05 and ***P < 0.001; ns, *P* > 0.10 using nonparametric Mann-Whitney testing.

Therefore, we focused on an alternative function of EPS8, which involves regulation of integrin signaling by altering endocytosis of integrin and epidermal growth factor receptor (EGFR) (*56*, *57*). Specifically, mammalian EPS8 constrains endocytosis of integrin and EGFR, and this inhibition is released upon EGF stimulation (*57*). A recent study found that hyperactivation of integrin signaling through several paradigms (growth on increased stiffness, constitutive active β1 integrin, hyperglycemia, etc.) results in mitochondrial fragmentation and robust UPR^MT^ activation through cytoskeletal remodeling (*14*). Consistent with these findings, we find that overexpression of β1 integrin in mammalian cells is sufficient to cause mitochondrial fragmentation and induce canonical UPR^MT^ targets (fig. S5, A and B). Moreover, knockdown of EPS8 using CRISPR interference resulted in increased integrin signaling as measured by phosphorylated focal adhesion kinase (pFAK) (fig. S5C). These studies were performed in MCF10A mammary epithelial cells grown on soft surfaces because a previous study showed that growth on plastic substrates were sufficient to drive integrin-mediated changes to mitochondria (*14*). Through these data, we hypothesized that a loss of *eps-8* can induce mitochondrial fragmentation and UPR^MT^ activation via an increase in integrin signaling. To test this hypothesis, we performed RNAi knockdown of the *C. elegans* homolog of β1 integrin, *pat-3*, and find that loss of *pat-3* robustly suppressed the induction of UPR^MT^ and the mitochondrial fragmentation observed in *eps-8* knockdown animals (Fig. 4). RNAi knockdown of *ina-1*, the homolog of α integrin, had no effects on UPR^MT^ or mitochondrial morphology. Furthermore, knockdown of *pat-3* resulted in a hyperfused mitochondrial network in the muscle and hypodermis, which further corroborate previous findings that integrin signaling is critical for regulating mitochondrial homeostasis (Fig. 4, C to F). Knockdown of *pat-3* was not sufficient to drive changes in mitochondrial morphology in the intestine, similar to *eps-8* knockdown, which provide further evidence that EPS-8 and PAT-3 alter mitochondrial morphology through the same mechanistic pathway (fig. S5D).

**Fig. 5.**
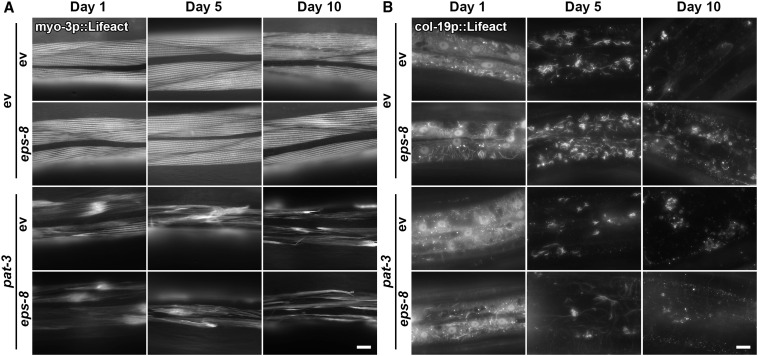
Loss of *eps-8* promotes cytoskeletal. (**A** and **B**) Representative fluorescent images of days 1, 5, and 10 adult animals expressing Lifeact::mRuby from a muscle-specific promoter *myo-3p* (A) or a hypodermal-specific promoter *col-19p* (B). Animals were grown on empty vector control or *eps-8* RNAi mixed in a 1:1 ratio with empty vector control or *pat-3* RNAi from hatch. Animals were aged on FUDR as described in Materials and Methods. Images were captured on a Zeiss AxioObserver Z1. Scale bar, 10μm.

Increased integrin signaling can alter many intracellular pathways to affect cell growth and has been shown to directly alter mitochondrial homeostasis through changes in the actin cytoskeleton. Specifically, hyperstabilization of actin filaments through ROCK (rho associated coiled-coil containing protein kinase) can induce mitochondrial fragmentation (*14*). Moreover, Koyuncu *et al.* (*53*) identified a role for EPS-8 in actin filament stability, such that RNAi knockdown of *eps-8* was sufficient to prevent an age-associated decline in myosin and actin filaments (*53*). Here, we directly tested the functional role of EPS-8 in actin cytoskeletal integrity using LifeAct, which robustly labels actin filaments in a tissue-specific manner (*58*). Consistent with findings by Koyuncu *et al.* (*53*), RNAi knockdown of *eps-8* was sufficient to delay an age-associated decline in cytoskeletal integrity in the muscle and hypodermis (Fig. 5). As previously described, actin in the muscle is visible as linear striations lining up along muscle filaments, which deteriorate during the aging process, whereas hypodermal actin is highly abundant at day 1 but reorganizes into actin-covered endocytic vesicles at mid-age (*58*). Knockdown of *eps-8* slowed the deterioration of actin filaments in the muscle and resulted in increased actin-covered vesicles at mid-age in the hypodermis. Perhaps, these changes in cytoskeletal dynamics were dependent on *pat-3*, similar to observed changes in mitochondrial homeostasis. While loss of *pat-3* itself caused defects in cytoskeletal integrity in both the muscle and hypodermis, the failure of *eps-8* knockdown to promote cytoskeletal integrity in animals lacking *pat-3* suggests that integrin functions downstream of EPS-8 to alter cytoskeletal function.

Since knockdown of *eps-8* results in increased stability of the cytoskeleton at late age, we next sought to determine whether loss of *eps-8* had a beneficial impact on life span. Similar to Koyuncu *et al.* (*53*), we do indeed find that loss of *eps-8* has a significant life-span extension (fig. S6). However, because we see that loss of *eps-8* directly affects mitochondrial homeostasis through integrin, we next studied whether the life-span extension is dependent on UPR^MT^ activation through integrin. Loss of the major activator of UPR^MT^, *atfs-1*, or *pat-3* fully suppressed the life-span extension found in these animals (fig. S6). Unexpectedly, simultaneous loss of *eps-8* and *pat-3* resulted in a decrease in life span, suggesting that without the increase in integrin signaling, loss of *eps-8* may actually have negative consequences.

Last, another hit from our screen *ZC239.5* is a predicted homolog of TNFAIP, which is a direct regulator of RhoA. Specifically, TNFAIP serves as a substrate-specific adaptor for the BTB-CUL3-RBX1 E3 ubiquitin-protein ligase complex and directly targets RHOA for ubiquitination. RhoA plays a role in promoting actin filament assembly (*59*) and is one of the direct targets downstream of integrin to hyperstabilize actin filaments (*14*). RNAi knockdown of *ZC239.5* phenocopies *eps-8* knockdown, resulting in increased UPR^MT^ induction and mitochondrial fragmentation (fig. S7). Although the effects of *ZC239.5* knockdown are milder than those exhibited by *eps-8* knockdown, this is consistent with a model whereby ZC239.5 and RhoA function downstream of integrin, which may have other functional targets of cytoskeletal remodeling beyond RhoA signaling (*14*).

## DISCUSSION

Overall, our cross-species screen serves as a unique platform bridging the regulation of mitochondrial homeostasis between mammals and *C. elegans*. Through CRISPR-Cas9 screening and transcriptome analysis in NPCs and screening of UPR^MT^, UPR^ER^, and HSR in *C. elegans*, we curated a large dataset that provides better understanding of conserved genes that regulate mitochondrial homeostasis and how they affect other stress responses. Specifically, we identified two regulators of the actin cytoskeleton that has direct implications in mitochondrial homeostasis: *eps-8* (EPS8/EPS8L), which alters integrin signaling to drive mitochondrial homeostasis through cytoskeletal remodeling. Together, our data support the hypothesis that loss of *eps-8* results in an integrin-mediated hyperstabilization of actin filaments, which can cause mitochondrial remodeling and UPR^MT^ activation. Our findings are exciting as the role of integrin signaling in mitochondrial remodeling and UPR^MT^, while previously described in mammals (*14*), has never been identified in *C. elegans*.

Still to be identified is how increased integrin signaling affects mitochondrial health. In mammals, increased β1 integrin signaling resulted in activation of ROCK, which altered calcium homeostasis in mitochondria to cause mitochondrial remodeling (*14*). Changes in mitochondrial structure resulted in activation of an OxSR through HSF-1 and NRF2, which results in increased stress resistance and survival in human cancer cells. It would be of interest to determine whether the functional output of EPS-8 in *C. elegans* similarly involves ROCK, HSF-1, and SKN-1 (NRF2 homolog) to enact actin and mitochondrial changes. Our screens also revealed a predicted homolog of TNFAIP, *ZC239.5*. TNFAIP is a direct regulator of RhoA serving as a substrate-specific adaptor for the BTB-CUL3-RBX1 E3 ubiquitin-protein ligase complex and directly targets RHOA for ubiquitination. RhoA plays a role in promoting actin filament assembly (*59*) and is one of the direct targets downstream of integrin to hyperstabilize actin filaments (*14*). Our screening data indicate that RNAi knockdown of *ZC239.5* phenocopies *eps-8* knockdown, resulting in increased UPR^MT^ induction, which is consistent with a model whereby ZC239.5 and RhoA function downstream of integrin to alter mitochondrial homeostasis.

A challenging question is whether the changes to mitochondria downstream of effects on actin are beneficial or detrimental. Previous studies have shown that mitochondrial dysfunction can cause activation of UPR^MT^ and have a beneficial hermetic effect (*6*). Moreover, the mitochondrial fragmentation observed in cancer cells downstream of integrin hyperactivation resulted in increased stress sensitivity in these cells due to activation of OxSRs (*14*). Last, a complementary study also identified EPS-8 and showed directly that loss of *eps-8* actually extends life span (*53*). These studies together with our finding beg the question of whether actin-mediated remodeling of mitochondria can be beneficial, despite causing mitochondrial fragmentation, due to the activation of beneficial stress responses. Further studies will help to shed light on the direct impact of EPS8-mediated cytoskeletal and mitochondrial remodeling in mammalian aging and disease.

## MATERIALS AND METHODS

### Culturing and maintaining NPCs

Human embryonic stem cell line H9 (*60*) was differentiated by double SMAD inhibition (*61*) into pan-lineage NPCs and passaged in Dulbecco’s modified Eagle’s medium (DMEM)/F-12 medium (Thermo Fisher Scientific, 11320082) supplemented with N2 (Thermo Fisher Scientific, 17502-048), B-27 minus Vitamin A (Thermo Fisher Scientific, 12587001), basic fibroblast growth factor (Thermo Fisher Scientific, PHG0026), glutamine (Thermo Fisher Scientific, 35050061), and penicillin/streptomycin (Thermo Fisher Scientific, 15070063). Unless otherwise stated, all NPC experiments were performed on cells grown in 4% oxygen and 5% carbon dioxide on plates coated with Geltrex (Thermo Fisher Scientific, A1413302) at a cell concentration of 2^6^ cells per 10-cm dish. At each passage, cells were plated with Rock inhibitor (Abcam, ab120129), which was removed when the medium was replaced after 1 day. Rotenone was diluted to a stock of 1.86 μg/μl concentration in ethanol (EtOH). Antimycin was diluted to a stock of 20 mM in EtOH.

### NPC rotenone screen

NPCs were transduced with lentivirus encoding Cas9 (pHK027-Blast) and selected with blasticidin. The surviving culture was expanded to 130 × 10^6^ cells and transduced with lentivirus containing the Avana sgRNA library pools 1 to 4 (*62*). This library contains four sgRNAs for each of 18,472 human genes and 663 sgRNAs of random sequence that are not expected to target human genes. The cells were selected using puromycin for 2 days and were experimentally shown to have a 56% survival rate. The cells were passaged for 2 weeks to allow editing to occur to completion and to allow the protein products time to deplete. At this point, cells were split into two arms—rotenone (3.5 ng/ml) or equal volume EtOH control—each maintaining at least 40 × 10^6^ cells for full library coverage. These arms were split as each became confluent and plated at 2 × 10^6^ cells per 15-cm dish. DNA was isolated and libraries were prepared according to (*63*).

### CellTiter-Glo luminescence viability assay

To validate the screen results, we generated individual lines of NPCs in which a single sgRNA (selected from the Avana4 library) and Cas9 were transduced, selected for, and grown out for 2 weeks to allow depletion of gene products. This strategy generates diverse pools of cells in which the gene targeted is expected to have a variety of mutations represented. For each experiment, cells were seeded in 96-well, black-walled plates (Corning, 3904) and grown overnight with rock inhibitor. The following day, medium was replaced with fresh medium lacking rock inhibitor but containing the final concentration of rotenone (0, 1.2, 2.3, 4.7, 9.4, 18.75, 37.5, 75, 150, 300, or 600 ng/ml). Following 3 days of stress, medium was removed by aspiration, and 50 μl of CellTiter-Glo (Promega, G7571)/media (1:9) was added to each well with a multichannel pipette. After an incubation of 30 min at room temperature, luminescence was measured using a Tecan M1000. Because of possible differences in baseline cell growth rates between Scramble-treated and sgRNA-treated, we plated two cell concentrations and chose the cell concentration for knockout and scramble with the most comparable growth for analysis. Except for the highest rotenone concentration, wells on the periphery of the plate were generally avoided. Values plotted are normalized to the untreated wells of each line. Error bars are normalized SD of three replicate wells. Two-tailed *t* tests were performed for the knockout (sgR) lines compared to the scramble (Scr) lines. sgRNA sequences are available in table S9.

### NPC antimycin treatment RNA-seq analysis

NPCs were plated in a medium containing rock inhibitor. The following day, the medium was replaced with medium-lacking rock inhibitor. Following an additional day of growth, cells were treated with 10 μM antimycin or equal volume of EtOH as vehicle control. Medium was aspirated, cells were collected in TRIzol (Thermo Fisher Scientific, 15596018) after 8 hours, mixed with equal volume of isopropanol, and RNA was extracted using RNeasy Mini Kit (QIAGEN, 74106).

RNA concentration from three biological replicates was measured using an Agilent Bioanalyzer at UC Berkeley Functional Genomics Core Lab (FGL). Total RNA (500 ng) was used for library construction using the mRNA HyperPrep Kit (Kapa Biosystems, KR1352). Compatible dual-indexed “barcode” adaptors (Kapa Biosystems, KR1318) were chosen so that all 10 libraries could be multiplexed on one lane on the Illumina HS4000 sequencer. Each library was run on the Agilent Bioanalyzer at the FGL for quality purposes. Libraries were submitted to the Vincent J. Coates Genomics Sequencing Core Laboratory for sequencing 150–base pair (bp) single-direction reads. The fastq files underwent adapter and low-quality score trimming. Libraries averaged ~108× transcriptome sequencing depth.

For isoform-level data (Fig. 1I), reads were mapped to hg37 using Salmon transcriptome analysis (https://usegalaxy.org/) with default parameters. With transcripts per million (TPM) mapped for each isoform, we decreased noise from low-expression isoforms by filtering out any isoforms with a TPM of <1 in any antimycin-treated sample and with a mean TPM of <1 across all antimycin- and EtOH-treated samples. Fold change for each isoform was calculated using (mean TPM for all three antimycin replicates)/(mean TPM for all three EtOH replicates). Any fold change of >2 was submitted for ATF5 binding site analysis. For improved visualization in Fig. 1I, we additionally filtered out any isoforms with a TPM of <1 in any sample and plotted mean antimycin TPM and mean EtOH TPM on log_2_ scales. These isoform data were aggregated to the gene level for visualization in Fig. 1G. For table S2, gene-level differential gene expression analysis, reads were mapped to the hg19 human reference genome and transcript sequences from Ensembl. The read counts were normalized by dataset before performing EDGE (Empirical Analyses of DGE, differential gene expression) analysis.

### ATF5 motif analysis

A window of 500 bp surrounding the Gencode-defined transcription start site(s) was analyzed for the presence of matches to PWM-represented (*64*) binding sites for known human transcription factors. We used the bedops software suite (*65*) for this purpose as follows. First, we extracted the relevant start site–surrounding sequence stretches from the human genome using the following parameters: bedops --range 500 -u /path/Gencode_tx-start.bed | grep -wf responsivegenes.txt > my_promoters.bed (where “Gencode_tx-start.bed” contains the genomic coordinates of transcription start sites, “responsivegenes.txt” is a list of names of genes that responded to the stimulus, and “promoters.bed” contains the sequences surrounding the start sites).

Next, we identified all matches to known transcription factor PWM binding sites using the following parameters: bedmap --echo-map my_promoters.bed /path/TF_PWMs.sort > promoter-TF-motifs.txt (where “TF_PWMs.sort” contains the locations of PWM consensus site matches for known human transcription factors in the human genome, “my_promoters.bed” contains promoter sequences of responsive genes, and “promoter-TF-motifs.txt” contains a gene-by-gene list of all PWM matches in those promoter sequences). To identify the list of genes to screen in *C. elegans*, we then retrieved from the twofold up-regulated isoform list, genes, the promoters of which contain a match to the ATF5 PWM (*66*).

### NPC RT-qPCR

Cells were plated and treated with 10 μM antimycin as above but for the indicated times. RNA was purified as above, and concentration was determined using a NanoDrop. One microgram of total RNA was reverse-transcribed using the QuantiTect Reverse Transcription Kit (QIAGEN, 205314), and 5 μl of complementary DNA (cDNA) was added to 5 μl of SYBR Select MasterMix (Thermo Fisher Scientific, 4472920) and 10 μM final primer concentration. Reactions were run in 384-well plates with 60°C annealing temperatures, and relative abundance was determined using a standard curve made from a mix of all cDNAs included in the experiment. Plotted are average fold changes of antimycin treatment/EtOH treatment within each time point of two to six replicates, with errors derived from SEM. Paired *t* tests were performed for each time point of the antimycin treatment compared to EtOH. Primer sequences are available in table S10.

### MCF10A cell culture

MCF10A cells were grown on 5 mM glucose DMEM/F-12 (1:1 mixture of F-12: 10 mM glucose and 0 mM glucose DMEM; Life Technologies, 11765054 and 11966025, respectively) supplemented with 5% horse serum (Gibco, 16051-122), epidermal growth factor (20 ng/ml; PeproTech), insulin (10 μg/ml; Sigma-Aldrich), hydrocortisone (0.5 μg/ml; Sigma-Aldrich), cholera toxin (100 ng/ml; Sigma-Aldrich, C8052-2MG), and 1× penicillin/streptomycin (Gibco). β1 cells were generated with a puromycin lentiviral transfer vector expressing 3× MYC-tagged β1 integrin using the tetracycline rtTA2(S)-M2 (*67*) inducible promoter. β1 integrin overexpression was accomplished by treating cells for 24 hours with doxycycline (200 ng/ml; Sigma-Aldrich, D9891).

### Biomimetic extracellular matrix–coated polyacrylamide hydrogel cell culture surfaces

Cleaned (10% ClO, 1 M HCl, and then 100% EtOH) round #1 German glass coverslips (Electron Microscopy Services) were coated with 0.5% (v/v) (3-aminopropyl)triethoxysilane (APTES; Sigma-Aldrich, 440140), 99.2% (v/v) EtOH, and 0.3% (v/v) glacial acetic acid for 2 hours and then cleaned in 100% EtOH on an orbital shaker at 22°C. APTES-activated coverslips were coated with phosphate-buffered saline (PBS)–buffered acrylamide/bis-acrylamide (Bio-Rad, 1610140 and 1610142) solution (3%/0.05%) polymerized with TEMED (tetramethylethylenediamine) (0.1%, v/v) (Bio-Rad, 1610801) and potassium persulfate (0.1%, w/v) (Thermo Fisher Scientific, BP180) to yield a final thickness of ~85 μm. Polyacrylamide (PA) gels were washed with 70% EtOH and sterile PBS before 3,4-dihydroxy-l-phenylalanine (DOPA) coating for 5 min at 22°C protected from light with sterile-filtered DOPA in pH 10 (10 mM) tris buffer (*68*). DOPA-coated PA gels were washed 2× with sterile PBS and extracellular matrix functionalized with human fibronectin (5 μg/ml; Millipore, FC010) in sterile PBS 1 hour at 37°C to generate an expected fibronectin-coating density of 6 μM/cm^2^.

### MCF10A qPCR

Total RNA was isolated from biological samples with TRIzol (Invitrogen, 15596-018) according to the manufacturer’s instructions. cDNA was synthesized with 1 μg of total RNA in 10 μl of reaction volume with RNA using M-MLV (Moloney Murine Leukemia Virus) reverse transcriptase (BioChain, Z5040002-100K) and 5× reaction buffer (BioChain, Z5040002-100K), random hexamers (Roche, 11034731001), dNTPs (deoxynucleoside triphosphate), and 1 U of RiboLock (Thermo Fisher Scientific, EO0384). RT-thermocycler program includes random hexamers and RNA incubated at 70°C for 10 min, then held at 4°C until the addition of the M-MLV reverse transcriptase, dNTPs, RiboLock, and M-MLV reverse transcriptase, then at 50°C for 1 hour, 95°C for 5 min, and then stored at −20°C until qPCR was performed. The reverse transcription reaction was then diluted to 50 μl of total volume with double-distilled H_2_O rendering a concentration of 20 ng of RNA per 1 μl used in subsequent qPCR reactions. qPCR was performed in triplicate using PerfeCTa SYBR Green FastMix (Quantabio, catalog no. 95072-05K) with an Eppendorf Mastercycler RealPlex2. qPCR thermocycler program includes 95°C for 10 min, then 40 cycles of a 95°C for 15 s, 60°C for 20 s, followed by a melt curve 60° to 95°C for more than 10 min. Melt curves and gel electrophoresis were used to validate the quality of amplified products. The Δ*C*_t_ values from independent experiments were used to calculate fold change of expression using the 2^−ΔΔ*C*t^ method. For each gene measured, the SEM of the Δ*C*_t_ values was calculated and used to generate positive and negative error values in the 2^−ΔΔ*C*t^ fold change space. Plots of qPCR data display bars representing the mean fold change + SEM and individual points representing the fold change value for each experiment relative to the mean. Primer sequences are available in table S10.

### MCF10A MitoTracker staining

MitoTracker deep red FM (Invitrogen, M22426) was solubilized in dimethyl sulfoxide to yield 100 μM frozen aliquots that were diluted into media to yield a 10 μM stock, which was added directly to cell culture media already in the culture yielding a final concertation of 100 nM (to prevent media change derived fluid flow shear stress) 30 min before 4% paraformaldehyde (PFA) fixation or live cell imaging (MitoTracker red FM was used for PFA-fixed samples). Samples were washed in 3× PBS and imaged with a Nikon Eclipse Ti spinning disc microscope, Yokogawa CSU-X, Andor Zyla sCMOS, Andor Multi-Port Laser unit, and Molecular Devices MetaMorph imaging suite.

### MCF10A actin and pFAK imaging

Cells were fixed in 4% PFA (Electron Microscopy Services, 15710) in 1× PBS for 30 min at room temperature, washed, and blocked with a blocking buffer (Hanks’ balanced salt solution fortified with 10% fetal bovine serum (HyClone), 0.1% bovine serum albumin (Thermo Fisher Scientific, BP1600), 0.05% saponin (EMD, L3771), and 0.1% Tween 20 (Thermo Fisher Scientific, BP337500). Primary antibodies (1:100 to 1:200) were applied for 2 hours at room temperature or 24 hours at 4°C, then secondary antibodies (1:1000) were applied for 2 hours at 22°C. We used Alexa Fluor 568 Phalloidin, Thermo Fisher Scientific A12380 and Cell Signaling Technologies pFAK (Tyr^397^), Antibody 3283 for staining.

### *C. elegans* strains and maintenance

All *C. elegans* strains are derivatives of the N2 wild-type worm from Caenorhabditis Genetics Center (CGC) and are listed in table S11. All worms were grown at 15° to 20°C on NGM (nematode growth media) agar plates as described in each specific experimental section. Worms were fed OP50 *Escherichia coli* B strain for general maintenance and switched to HT115 *E. coli* K12 strain after synchronization for all experimentation. HT115 bacteria were carrying the pL4440 empty vector control or expressing double-stranded RNA containing the sequence against a target gene. All experiments were performed on age-matched animals synchronized using a standard bleaching protocol. Briefly, animals were harvested with M9 solution (22 mM KH_2_PO_4_ monobasic, 42.3 mM Na_2_HPO_4_, 85.6 mM NaCl, and 1 mM MgSO_4_), bleached using a solution of 1.8% sodium hypochlorite and 0.375 M KOH diluted in M9 until all carcasses were digested. Intact eggs were then washed 4× with M9 solution.

### *C. elegans* screen and stereoscope imaging of reporters

*C. elegans* orthologs of human genes were identified using OrthoList 2 (*24*). RNAis were isolated from the Vidal or Ahringer RNAi libraries and sequence-verified using standard Sanger sequencing. Only RNAi constructs that matched the expected sequences were used for screening. For UPR^MT^, *hsp-6p::GFP* animals were grown on standard RNAi plates from hatch at 20°C until day 1 of adulthood. Animals were grown on 100% candidate RNAi for basal induction of UPR^MT^ or for stress induction of UPR^MT^. Candidate RNAi (80%) was mixed with 20% of either *cco-1* or *nuo-4* RNAi. For UPR^ER^ measurements, *hsp-4p::GFP* animals were grown on standard RNAi plates from hatch at 15°C until the L4 stage. Animals were then washed off with M9 and moved onto equivalent RNAi plates containing tunicamycin (25 ng/μl) and grown at 20°C for 24 hours. For HSR, *hsp-16.2p::GFP* animals were grown on standard RNAi plates from hatch at 20°C until day 1 of adulthood. Animals were then heat-shocked at 34°C for 2 hours and recovered at 20°C for 2 hours.

Initially, we performed screening by qualitatively measuring the level of *hsp-6p::GFP* expression by eye. To increase the objectivity of a visual screen, we performed two independent biological replicates of the screen, with reporter gene activity being assessed by two individual experimenters blinded to the identity of the RNAis. The induction of the reporter was scored on an integer scale from −3/0/+3 relative to wild-type animals grown on an empty vector control (0) or *cco-1* or *nuo-4* RNAi (+3). To measure suppression of stress-induced UPR^MT^ activation, the induction of the reporter was scored on a similar scale where *cco-1* or *nuo-4* RNAi knockdown was set as our baseline 0 point and loss or increase in this GFP signal is scored from −3/0/+3. Moreover, physiological phenotypes, including defects in development, egg-laying, and overall health, were reported. Last, a general assessment of fecundity was performed on an integer scale from −3/0/+3 based on the number of progeny visible on the plate at the time of measurement, as defects in mitochondrial function have direct impacts on fecundity (*69*). Similar to *hsp-6p::GFP* induction, fecundity measurements were performed relative to a wild-type control grown on empty vector and against *cco-1* or *nuo-4* RNAi. Similar integer scales were used for *hsp-4p::GFP* and *hsp-16.2p::GFP*, and raw screening data are available in tables S5 and S6. Top hits were screened for a third replicate, where all animals were imaged under a stereoscope and raw images are provided in the Supplementary Materials. A similar integer scale of −3/0/+3 were used to provide semiquantitative data of all images and are provided in tables S5 and S6.

For imaging of fluorescent reporters, staged worms were picked under a standard dissection microscope under white light at random to avoid biased sampling. Worms were immobilized in 100 nM sodium azide and aligned on a solid NGM plate. Images were captured on a Leica M250FA stereoscope equipped with a Hamamatsu ORCA-ER camera driven by LAS-X software.

### *C. elegans* wide-field and Airyscan microscopy

Animals were prepared for live-cell, high-resolution microscopy by staging using a standard bleaching protocol described above and maintaining animals on the specified RNAi until the desired aging. For standard experiments on young adults, all imaging experiments were performed at day 1 of adulthood. For all aging experiments, animals were aged by maintaining animals on RNAi plates supplemented with FUDR (5-fluoro-2′-deoxyuridine) starting from day 1 of adulthood until the desired age. FUDR plates were prepared by spotting the bacterial lawn with 100 μl of FUDR (10 mg/ml). For live-cell imaging, individual worms were picked off plates and mounted directly onto a microscopy slide containing M9. A coverslip was placed onto the sample and sealed with nail polish and imaged immediately for a maximum of 5 min. For standard wide-field microscopy, images were acquired on a Zeiss AxioObserver Z1 microscope equipped with a Lumencor SOLA light engine and a Zeiss Axiocam 506 camera driven by Zeiss ZenBlue software using a 63×/1.4 Plan Apochromat objective and standard GFP filter (Zeiss filter set 46 HE) or a dsRed filter (Zeiss filter set 43); or on a Leica DM6000 equipped with a Lumencor SOLA light engine, 63×/1.4 Plan Apochromat objective, standard GFP filter (Leica filter set 11504164), and a DFC9000 camera, driven on LAS-X software. For high-resolution microscopy, images were acquired on a Zeiss AxioObserver 7 LSM900 Airyscan 2 equipped with a 63×/1.4 Plan Apochromat objective, MA–photomultiplier tube (PMT) detector, diode lasers (488 nm, 10 mW, laser class 3B; 561 nm, 10 mW, laser class 4B), driven by ZenBlack software. Images were processed using ZEN Module Airyscan for three dimensions using default software settings. For quantification, images were analyzed using MitoMAPR_Batch (*70*). For muscle mitochondria, single-slice images were used, and analysis was performed by cropping single muscle cells. Two to three muscle cells were quantified across 6 to 10 worms per sample. For the hypodermis, max projection images were used, and analysis was performed across entire images on 6 to 10 worms per sample.

### *C. elegans* biosorter analysis

For quantitative analysis of fluorescent reporters, a Union Biometrica complex object analysis sorter bioSorter (product no. 250-5000-000) was used as previously described (*25*). Briefly, staged worms are washed off plates using M9, and signal is collected using a 488-nm light source. Data are represented as an integrated intensity of fluorescence normalized to the size of the animal using the integrated GFP output and dividing by the integrated extinction output. All data that exceed the measurement capacity of the PMT, calculated as a signal of 65355, are considered saturated and are censored from the calculation. Nonparametric Mann-Whitney testing was used in Prism8 software to obtain *P* values.

### *C. elegans* qPCR and RNA-seq analysis

Animals were synchronized using a standard bleaching protocol and all RNA collection was performed at day 1 of adulthood. A total of ~1000 animals were harvested using M9, and animals were pelleted by gravity by allowing worms to settle to the bottom of the tube over 2 min. M9 was subsequently aspirated and replaced with TRIzol solution, worms were freeze-thawed 3× with liquid nitrogen, and a ~30-s vortexing was performed before each refreeze. After the final thaw, chloroform was added at a 1:5 ratio (chloroform:TRIzol), and aqueous separation of RNA was performed via centrifugation in a heavy gel phase-lock tube (VWR, 10847--802). The aqueous phase was collected, mixed with isopropanol at a 1:1 ratio, and then RNA purification was performed using a QIAGEN RNeasy Mini Kit (74106) as per the manufacturer’s directions.

For RNA-seq analysis, library preparation was performed using Kapa Biosystems mRNA Hyper Prep Kit. Sequencing was performed at the Vincent J. Coates Genomic Sequencing Core at the University of California, Berkeley using an Illumina HS4000, mode SR100. Analysis was performed on the CLC Workbench v7.0.3. Mapping was performed using the Ensembl/WormBase (PR513758.WS273) *C. elegans* reference genome and transcript. Unique reads were calculated for each library, all data were normalized for the number of reads per library, adding a factor of 0.01 to eliminate zero values, and each expression value was transformed by log_2_. Four biological replicates were compiled per condition to run EDGE testing to provide significantly up-regulated or down-regulated genes per sample.

For qPCR, cDNA synthesis was performed using the QIAGEN QuantiTect Reverse Transcription Kit (205311) using 1 μg of RNA. Subsequent qPCR was performed using a general standard curve protocol with SYBR Select Master Mix (Life Technologies, 4472920), using a pool of all cDNA samples in equal ratios as a template for the standard curve of each primer pair. Four technical replicates were performed for each of three biological replicates per sample. Student *t* test was performed to calculate *P* values.

### *C. elegans* seahorse analysis

Synchronized animals were collected from plates using M9. Adult animals were isolated by settling adult worms with gravity in M9 for 2 min and aspirating off eggs/L1s in the M9 solution. Animals were washed three times using this method to clarify any bacteria and remove all eggs/L1 from the worm/M9 mix. Ten worms in M9 were placed into each well of a Seahorse XF96 cell culture microplate. Basal oxygen consumption rate was measured using an XFe96 sensor cartridge on the Seahorse XFe96 Analyzer with 2-min mixing, 30-s waiting, and 2-min measuring.

## Supplementary Materials

### This PDF file includes:

Figs. S1 to S7 Legends for tables S1 to S11 Supplemental files S1 to S25 References

## Notes

View/request a protocol for this paper from *Bio-protocol*.
