# Supplementary material for: Cross-species screening platforms identify EPS-8 as a critical link for mitochondrial stress and actin stabilization
Source: Sci Adv. 2021 Oct 29;7(44):eabj6818. doi: 10.1126/sciadv.abj6818 (PMC8555897; doi:10.1126/sciadv.abj6818)
Supplement: Supplementary file 1 — Figs. S1 to S7 Legends for tables S1 to S11 Supplemental files S1 to S25 References [file sciadv.abj6818_sm.pdf]

Science Advances
AAAS

Supplementary Materials for

Cross-species screening platforms identify EPS-8 as a critical link for mitochondrial stress and actin stabilization

Erica A. Moehle, Ryo Higuchi-Sanabria, C. Kimberly Tsui, Stefan Homentcovschi, Kevin M. Tharp, Hanlin Zhang, Hannah Chi, Larry Joe, Mattias de los Rios Rogers, Arushi Sahay, Naame Kelet, Camila Benitez, Raz Bar-Ziv, Gilberto Garcia, Koning Shen, Phillip A. Frankino, Robert T. Schinzel, Ophir Shalem, Andrew Dillin*

*Corresponding author. Email: dillin@berkeley.edu

Published 29 October 2021, *Sci. Adv.* **7**, eabj6818 (2021)
DOI: 10.1126/sciadv.abj6818

The PDF file includes:

Figs. S1 to S7
Legends for tables S1 to S11
Supplemental files S1 to S25
References

Other Supplementary Material for this manuscript includes the following:

Tables S1 to S11

Supplementary Figures, Tables, and Legends.

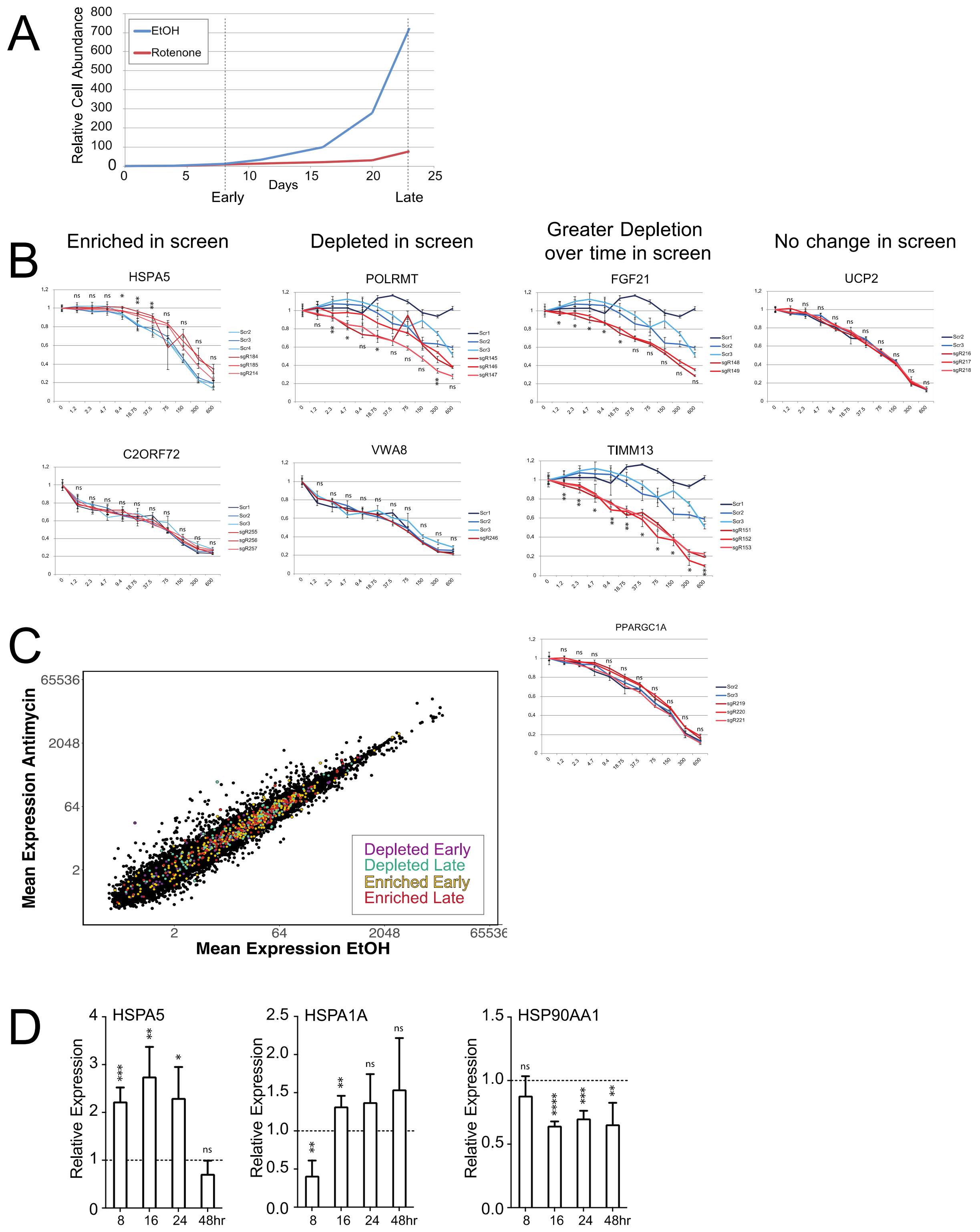

Fig. S1. Validation of additional targets from NPC screens. (A) Cell growth was monitored during the screen by performing duplicate cell counts with Trypan Blue and a Countess Cell

Counter. Only viable cells were included. A fraction of each was replated at the appropriate density. This fraction and the cell count at each split were propagated to calculate the total cell counts expected if all cells had been replated, relative to the cell count at the first split. Time points of collection are indicated as “Early” and “Late”. **(B)** Cells growth plotted as in **Fig. 1D**, where the x-axis denotes rotenone concentration (0, 1.2, 2.3, 4.7, 9.4, 18.75, 37.5, 75, 150, 300 and 600 ng/mL) and the y-axis indicates relative luminescence of each concentration compared to 0 ng/ml rotenone. The column headers indicate the behavior of each gene in the screen. Note that several genes did not reproduce the expected growth phenotype. **(C)** Mean expression is plotted as in **Fig. 1G**, in transcripts from individual genes (in transcripts per million). All transcripts of genes identified in the rotenone screen are highlighted in the specified colors. **(D)** RT-qPCR following treatment of NPCs with 10uM Antimycin, as in **Fig. 1D**. Mapped are fold change for 2-6 biological replicates of antimycin treatment to ethanol treatment with SEM and paired t-test for each timepoint (ns (not significant), * (<0.05), ** (<0.01), *** (<0.001), **** (<0.0001)).

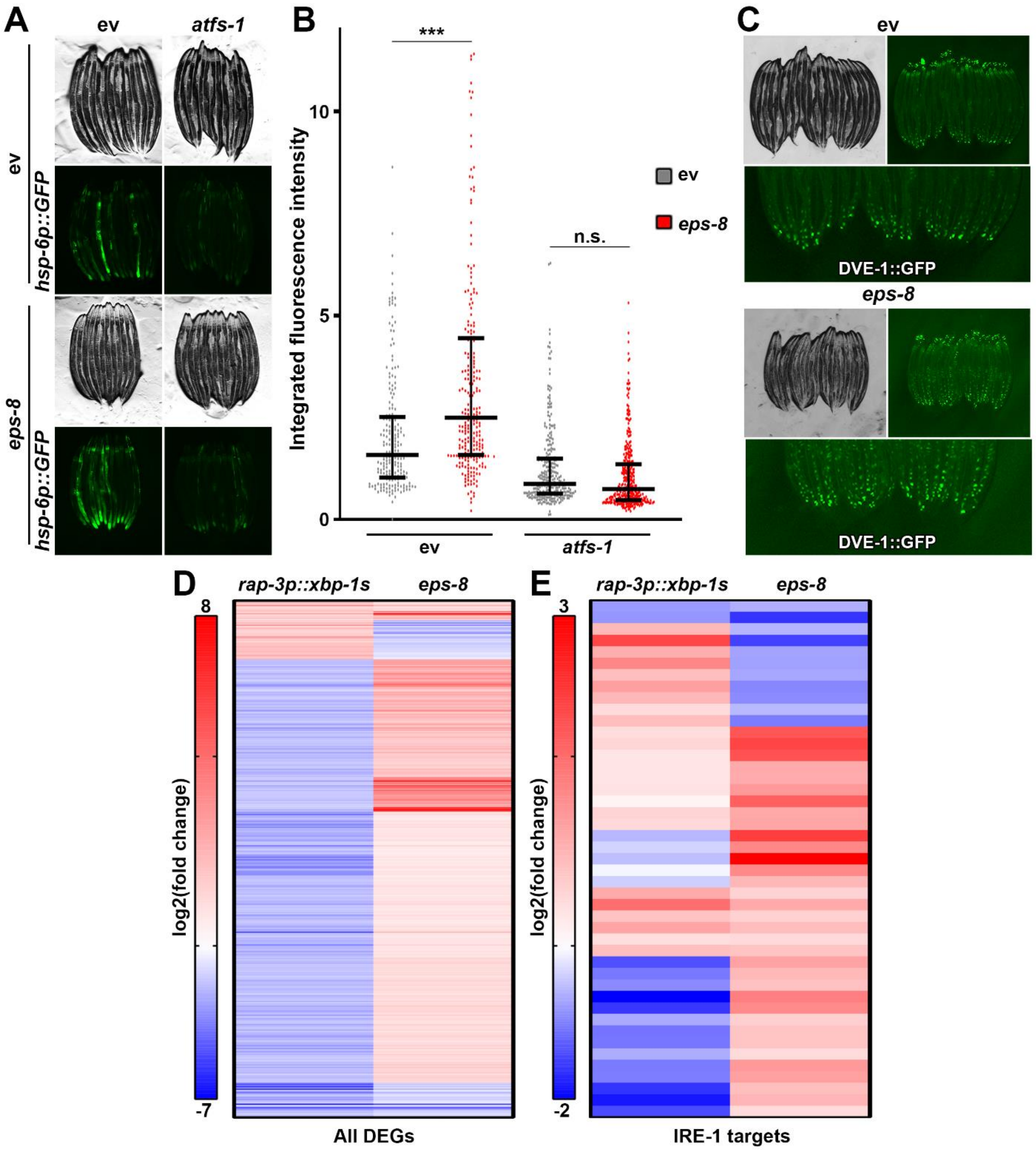

Fig. S2. Loss of *eps-8* induces a canonical UPR^{MT} signature. (A) Fluorescent micrographs of day 1 adult *hsp-6p::GFP* animals grown on empty vector control (ev) or *eps-8* RNAi mixed in 1:1 ratio with ev or *atfs-1* RNAi from hatch. All images are contrast matched. **(B)** Quantification of *hsp-6p::GFP* in day 1 adult animals grown on ev (grey) or *eps-8* RNAi (red) mixed in 1:1 ratio with ev or *atfs-1* RNAi from hatch. Lines represent median and interquartile range, with each dot representing a single animal. n = 206-332 per sample. Data is representative of 3 independent trials. *** = $p < 0.001$; n.s. = nonsignificant, $p > 0.10$ using non-parametric Mann-Whitney testing. **(C)** Fluorescent micrographs of day 1 adult *DVE-1::GFP* animals grown on empty vector control (ev) or *eps-8* RNAi from hatch. All images are contrast matched. Bottom panel shows a zoomed in version of the bottom half of the fluorescent image for better clarity. **(D)** RNA-seq

was performed in N2 animals grown on ev or *eps-8* RNAi as described in Materials and Methods. Heat map indicates log2(fold change) of genes in comparison to control where red indicates upregulated genes and blue indicates downregulated genes. Here, canonical UPR^{ER} genes are represented as those that were differentially expressed by neuronal overexpression of *xbp-1s* compared to an ev control as previously described (*49*). See Supplementary Table 5 for actual values of log2(fold change). **(E)** Differentially expressed genes from **(d)** that were previously identified as bonafide IRE-1 targets were extracted and plotted separately. IRE-1 targets were defined as genes that showed decreased expression when *ire-1* was mutated (*73*) and/or are part of the GO term 0030968 endoplasmic reticulum unfolded protein response using BioMart WormBase Parasite.

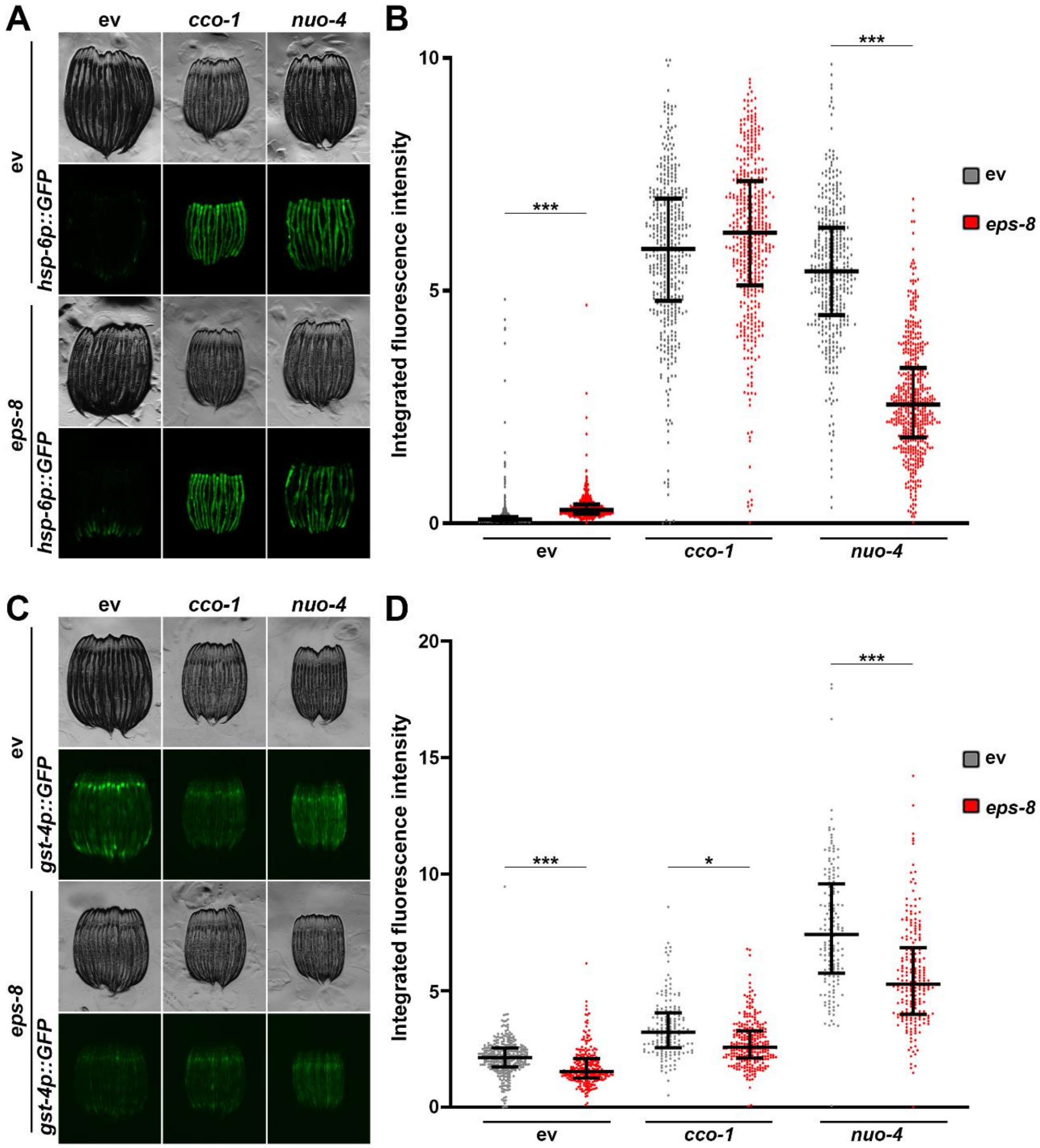

Fig. S3. Loss of *eps-8* suppresses induction of UPR^{MT} via inhibition of complex I, but not complex IV. (A) Fluorescent micrographs of day 1 adult *hsp-6p::GFP* animals grown on empty vector control (ev) or *eps-8* RNAi mixed in 1:1 ratio with ev, *cco-1*, or *nuo-4* RNAi from hatch. All images are contrast matched. **(B)** Quantification of *hsp-6p::GFP* in day 1 adult animals grown on ev (grey) or *eps-8* RNAi (red) mixed in 1:1 ratio with ev, *cco-1*, or *nuo-4* RNAi from hatch. Lines represent median and interquartile range, with each dot representing a single animal. n = 395-483 per sample. Data is representative of 3 independent trials. *** = $p < 0.001$ using non-parametric Mann-Whitney testing. **(C)** Fluorescent micrographs of day 1 adult *gst-4p::GFP* animals grown on empty vector control (ev) or *eps-8* RNAi mixed in 1:1 ratio with ev, *cco-1*, or *nuo-4* RNAi from hatch. All images are contrast matched. **(D)** Quantification of *gst-4p::GFP* in day 1 adult animals grown on ev (grey) or *eps-8* RNAi (red) mixed in 1:1 ratio with ev, *cco-1*, or *nuo-4* RNAi from hatch. Lines represent median and interquartile range, with each dot representing a single animal. n = 153-319 per sample. Data is representative of 3 independent trials. *** = $p < 0.001$ using non-parametric Mann-Whitney testing.

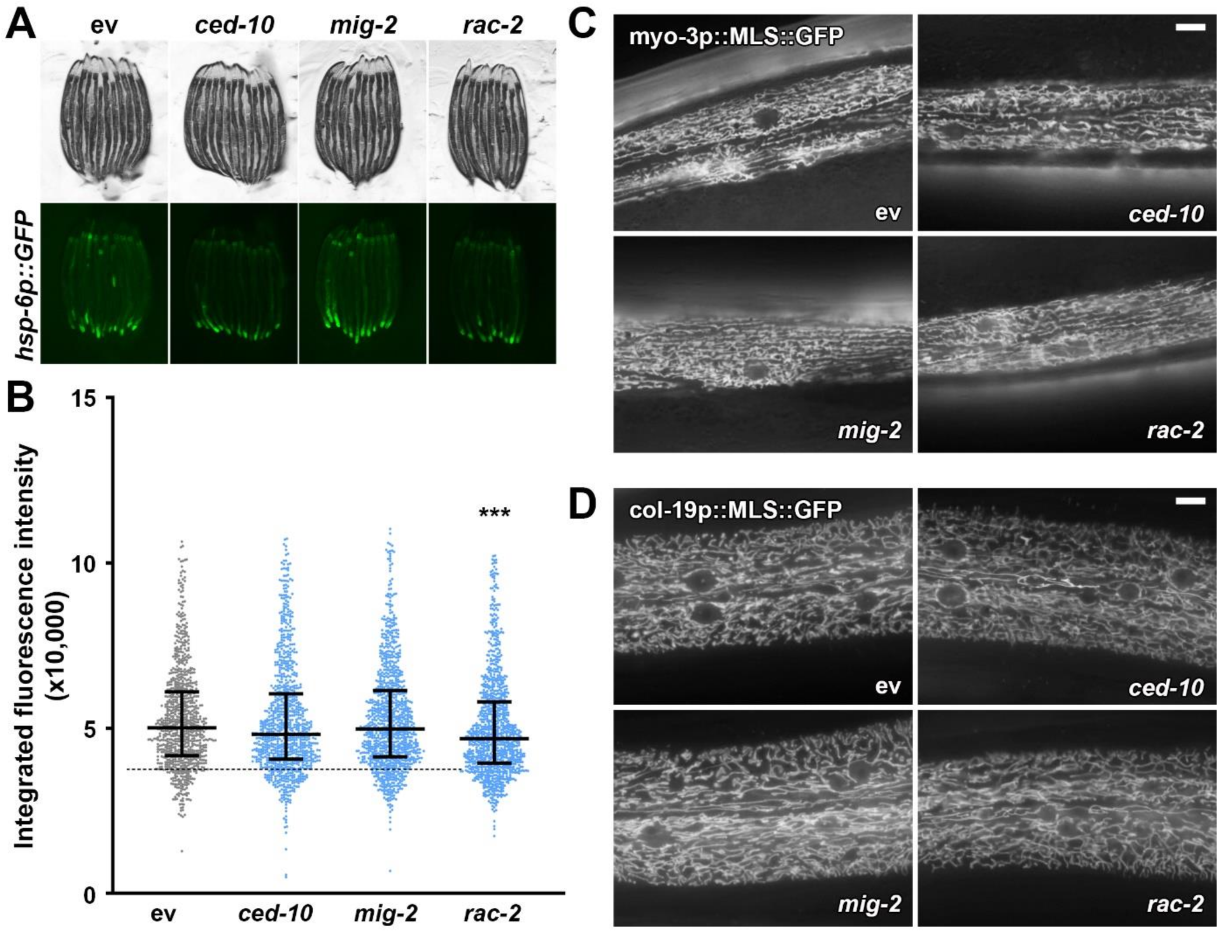

Fig. S4. Decreased RAC signaling does not alter mitochondrial homeostasis. (A) Fluorescent micrographs of day 1 adult *hsp-6p::GFP* animals grown on empty vector control (ev), *ced-10*, *mig-2*, or *rac-2* RNAi from hatch. All images are contrast matched. **(B)** Quantification of *hsp-6p::GFP* in day 1 adult animals grown on ev (grey), *ced-10*, *mig-2*, or *rac-2* (blue) RNAi from hatch. Lines represent median and interquartile range, with each dot representing a single animal. n = 927-1050 per sample. Data is representative of 3 independent trials. *** = $p < 0.001$ using non-parametric Mann-Whitney testing. **(C-D)** Representative fluorescent images of day 1 adult animals expressing a mitochondria-targeted GFP from tissue specific promoters: *myo-3p* (muscle, c) and *col-19p* (hypodermis, d). Animals were grown on ev, *ced-10*, *mig-2*, or *rac-2* RNAi from hatch and imaged directly on glass slides as described in Materials and Methods. Images were captured on a Leica DM6000. Scale bar is 10 µm.

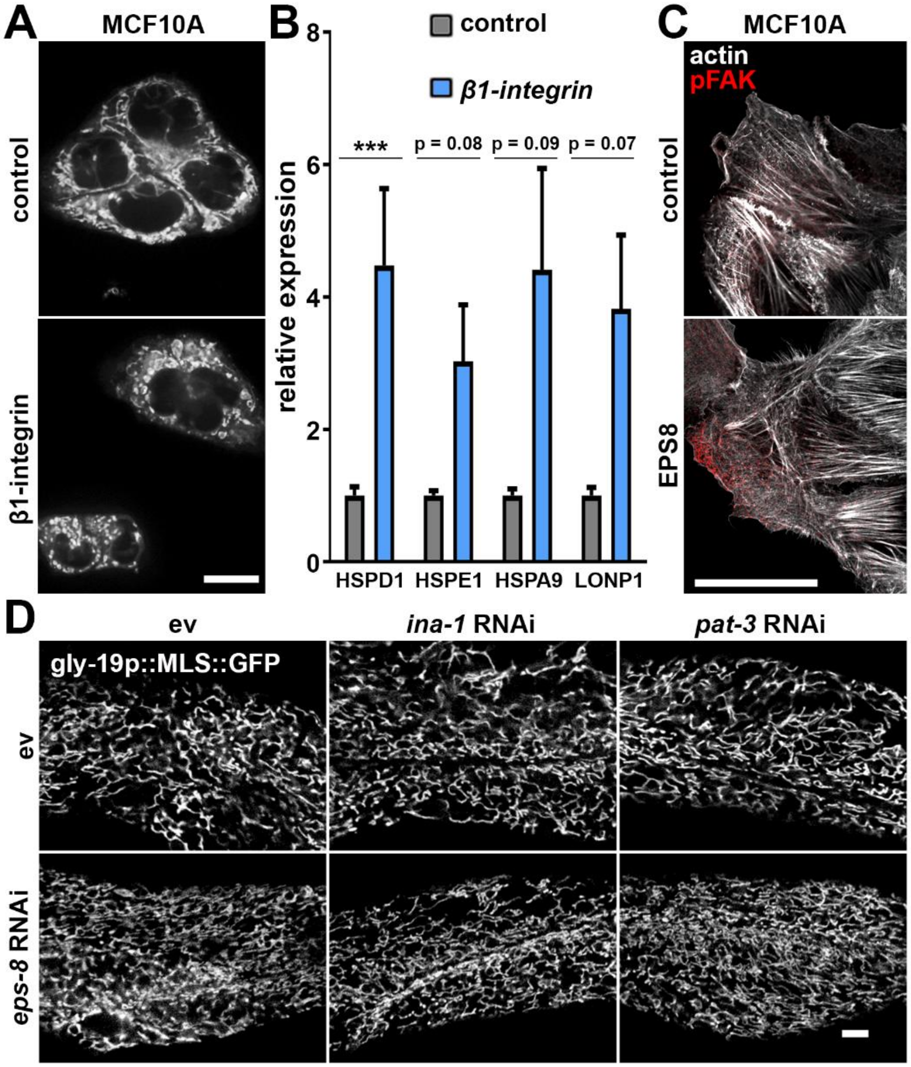

Fig. S5. Integrin signaling alters mitochondrial homeostasis. (A) Fluorescent micrographs of MCF10A cells expressing 3x-MYC-tagged β1-integrin from a tetracycline rtTA2(S)-M2 inducible promoter as described in Materials and Methods. Control images are cells without induction, and β1 integrin overexpressing cells are treated with 200 ng/mL doxycycline for 24 hours prior to imaging. Mitochondria are visualized by staining with mitotracker deep red FM as described in Materials and Methods using a Nikon Eclipse Ti spinning disc microscope. Scale bar is 10 µm. **(B)** qPCR was performed on MCF10A cells expressing 3x-MYC-tagged β1-integrin from a tetracycline rtTA2(S)-M2 inducible promoter. β1-integrin overexpression was induced by treatment with 200 ng/mL doxycycline for 24 hours prior to RNA collection. Data is presented as mean +/- SEM and is representative of 3 biological replicates. **(C)** Fluorescent micrographs of MCF10A cells transduced with lentiviral vectors carrying CRISPRi guides against a non-coding region of ACOC2 (control) or EPS8. Cells were fixed with 4% paraformaldehyde for 30 min and stained with Alexa Fluor 568 to visualized actin (white) and antibodies against pFAK (red) as described in Materials and Methods. Scale bar is 10 µm. **(D)** Representative fluorescent images of day 1 adult animals expressing a mitochondria-targeted GFP from an intestinal specific promoter, *gly-19p*. Animals were grown on ev or *eps-8* RNAi mixed in a 1:1 ratio with ev, *ina-1*, or *pat-3* RNAi from hatch and imaged directly on glass slides as described in Materials and Methods. Images were captured on an LSM900 Airyscan microscope. Scale bar is 10 µm.

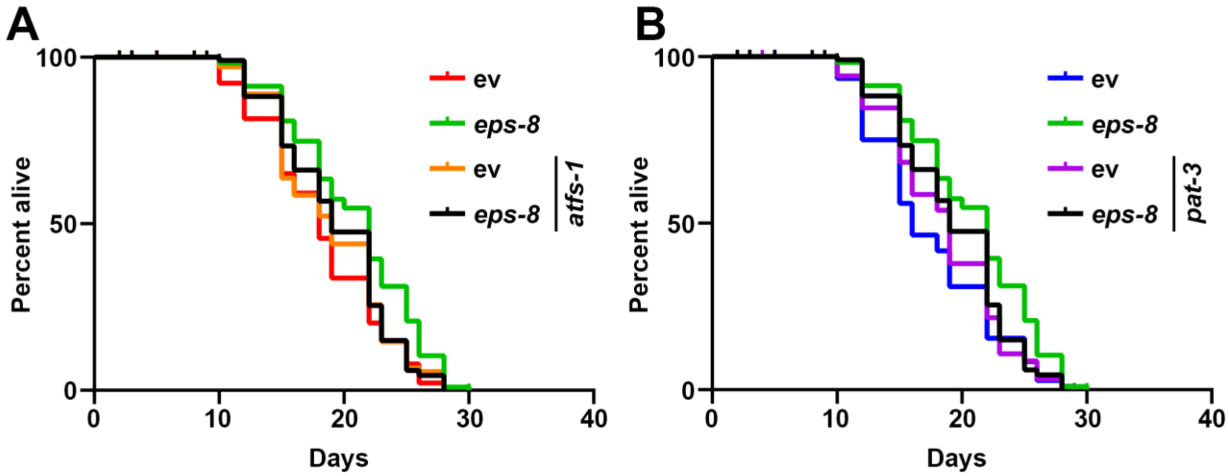

Fig. S6. Loss of *eps-8* extends lifespan in a UPR^{MT}-dependent manner.
(**A**) Lifespans of wild-type animals grown on control (ev) or *eps-8* RNAi mixed 50/50 with ev (red and green) or *atfs-1* (orange and black) RNAi from hatch. (**B**) Lifespans of wild-type animals grown on control (ev) or *eps-8* RNAi mixed 50/50 with ev (blue and green) or *pat-3* (purple and black) RNAi from hatch. Data for A and B were collected simultaneously and can be directly compared and were separated for ease of visibility. Statistics are as follows: (median survival, p-value using log-rank compared to ev, # of animals, % censored) ev (19, n/a, 120, 26%), *atfs-1* (19, 0.755, 120, 21%), *pat-3* (19, 0.18, 120, 18%), *eps-8* (22, 0.002, 120, 8%), *eps-8/atfs-1* (18, 0.883, 120, 18%), *eps-8/pat-3* (16, 0.001, 20%). Data is representative of 3 independent trials.

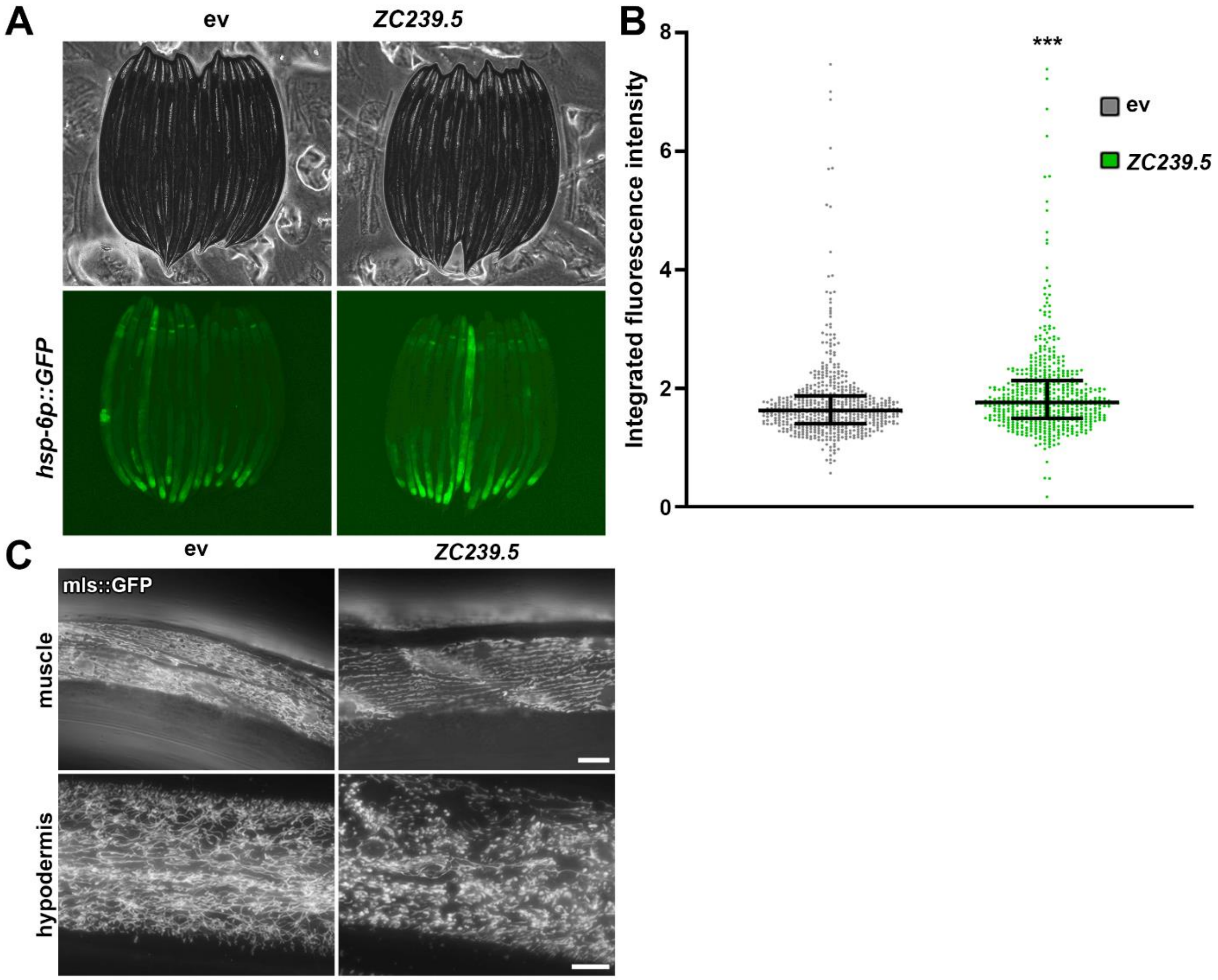

Fig. S7. Loss of *ZC239.5* similarly alters mitochondrial homeostasis to *eps-8* knockdown. (A) Fluorescent micrographs of day 1 adult *hsp-6p::GFP* animals grown on empty vector control (ev) or *ZC239.5* RNAi from hatch. All images are contrast matched. **(B)** Quantification of *hsp-6p::GFP* in day 1 adult animals grown on ev (grey) or *ZC239.5* (green) RNAi from hatch. Lines represent median and interquartile range, with each dot representing a single animal. n = 579 for ev and 555 for *ZC239.5*. Data is representative of 3 independent trials. *** = $p < 0.001$ using non-parametric Mann-Whitney testing. **(C)** Representative fluorescent images of day 1 adult animals expressing a mitochondria-targeted GFP from tissue specific promoters: *myo-3p* (muscle) and *col-19p* (hypodermis). Animals were grown on ev or *ZC239.5* RNAi from hatch and imaged directly on glass slides as described in Materials and Methods. Muscle images were captured on a standard wide-field Zeiss AxioObserver.Z1. Hypodermal images were captured on an LSM900 Airyscan microscope. Scale bar is 10 µm.

Table S1. NPC CRISPR-Cas9 screen gene list. Raw data of CRISPR-Cas9 screen providing all genes identified in the screen.

Table S2. NPC RNA-seq gene list. Raw data of NPC RNA-seq providing all genes sequenced.

Table S3. *C. elegans* screen gene list. List of human genes that were followed up in *C. elegans* secondary screens. Homologies were identified using Ortholist 2.

Table S4. *C. elegans* screen data. Raw *C. elegans* screening data for all genes for 2 biological replicates. Red indicates decreased GFP expression or decrease in fecundity (egg count), yellow indicates no change, and green indicates an increase in GFP expression or increase in fecundity (egg count) qualitatively by eye. Any visible phenotypes were also recorded.

Table S5. *C. elegans* Basal screen data. Raw numerical data for **Fig. 2B**. Also provides locations for each image in the supplementary images.

Table S6. *C. elegans* Suppressor screen data. Raw numerical data for **Fig. 2C**. Also provides locations for each image in the supplementary images.

Table S7. RNAseq *eps-8* vs. *cco-1* RNAi. Raw numerical data for **Fig. 3C**.

Table S8. RNAseq *eps-8* RNAi vs. *rab-3p::xbp-1s*. Raw numerical data for **Fig. 3D**.

Table S9. sgRNAs used in this study. sgRNA sequences used in this study are provided in this table.

Table S10. Primers used in this study. All primer sequences used in this study are provided in this table.

Table S11. Strains used in this study. All *C. elegans* strains used in this study are derivatives of the Bristol N2 strain and are detailed in this table.

Supplemental Information – Raw Screen Images

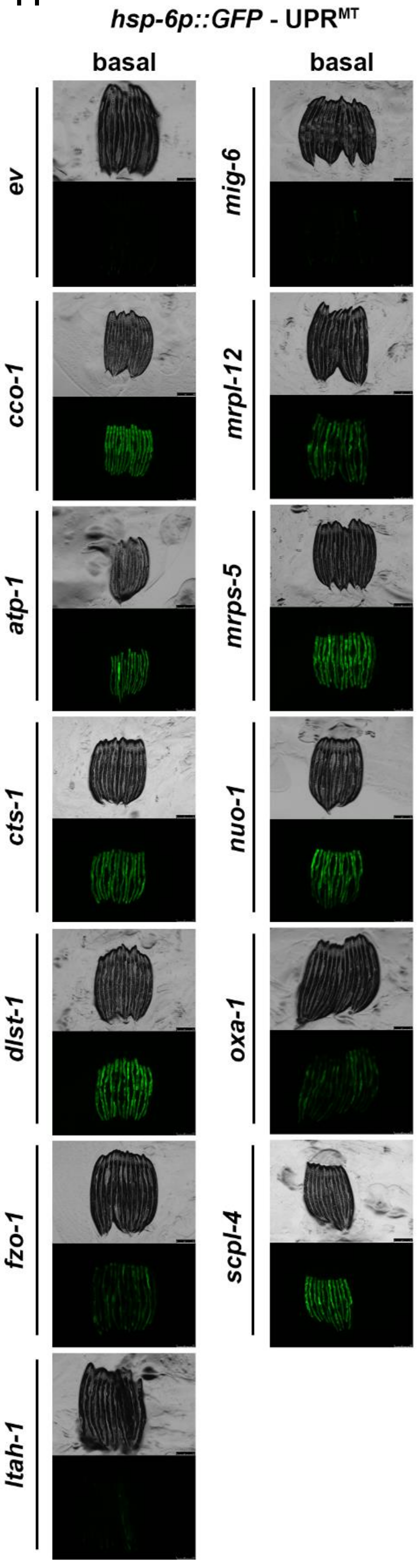

Supplemental File 1. UPR^{MT} Screen. *hsp-6p::GFP* animals were grown on RNAi indicated from hatch and imaged at day 1 of adulthood.

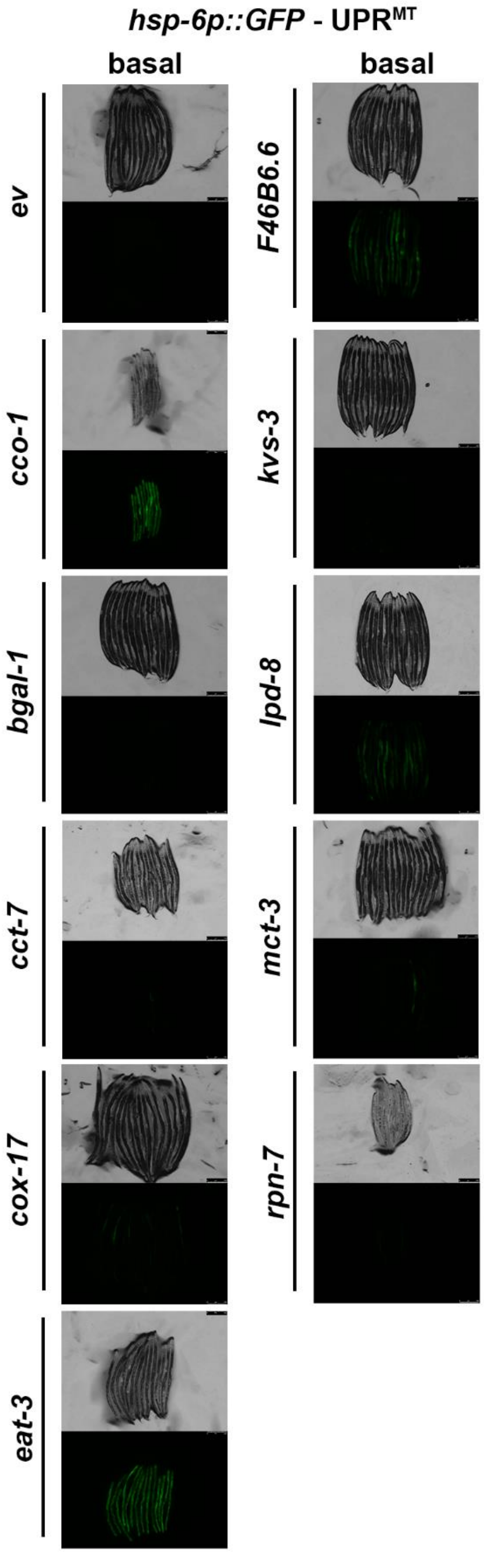

Supplemental File 2. UPR^{MT} Screen. *hsp-6p::GFP* animals were grown on RNAi indicated from hatch and imaged at day 1 of adulthood.

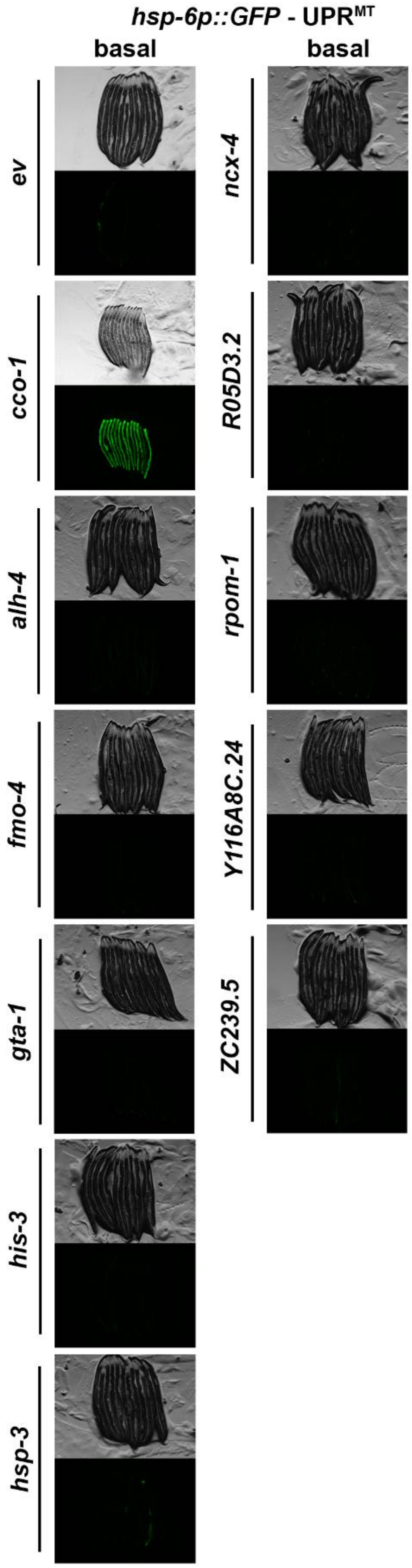

Supplemental File 3. UPR^{MT} Screen. *hsp-6p::GFP* animals were grown on RNAi indicated from hatch and imaged at day 1 of adulthood.

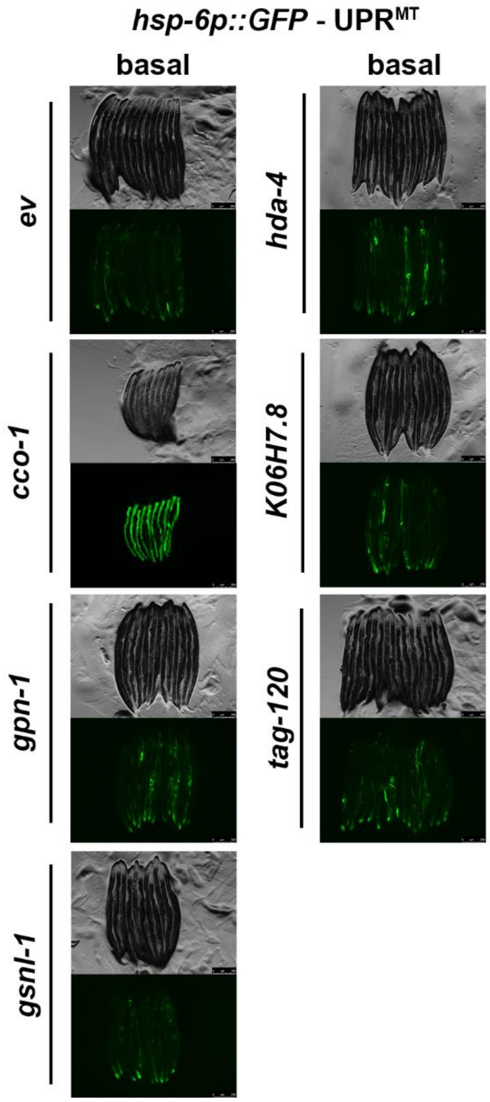

Supplemental File 4. UPR^{MT} Screen. *hsp-6p::GFP* animals were grown on RNAi indicated from hatch and imaged at day 1 of adulthood.

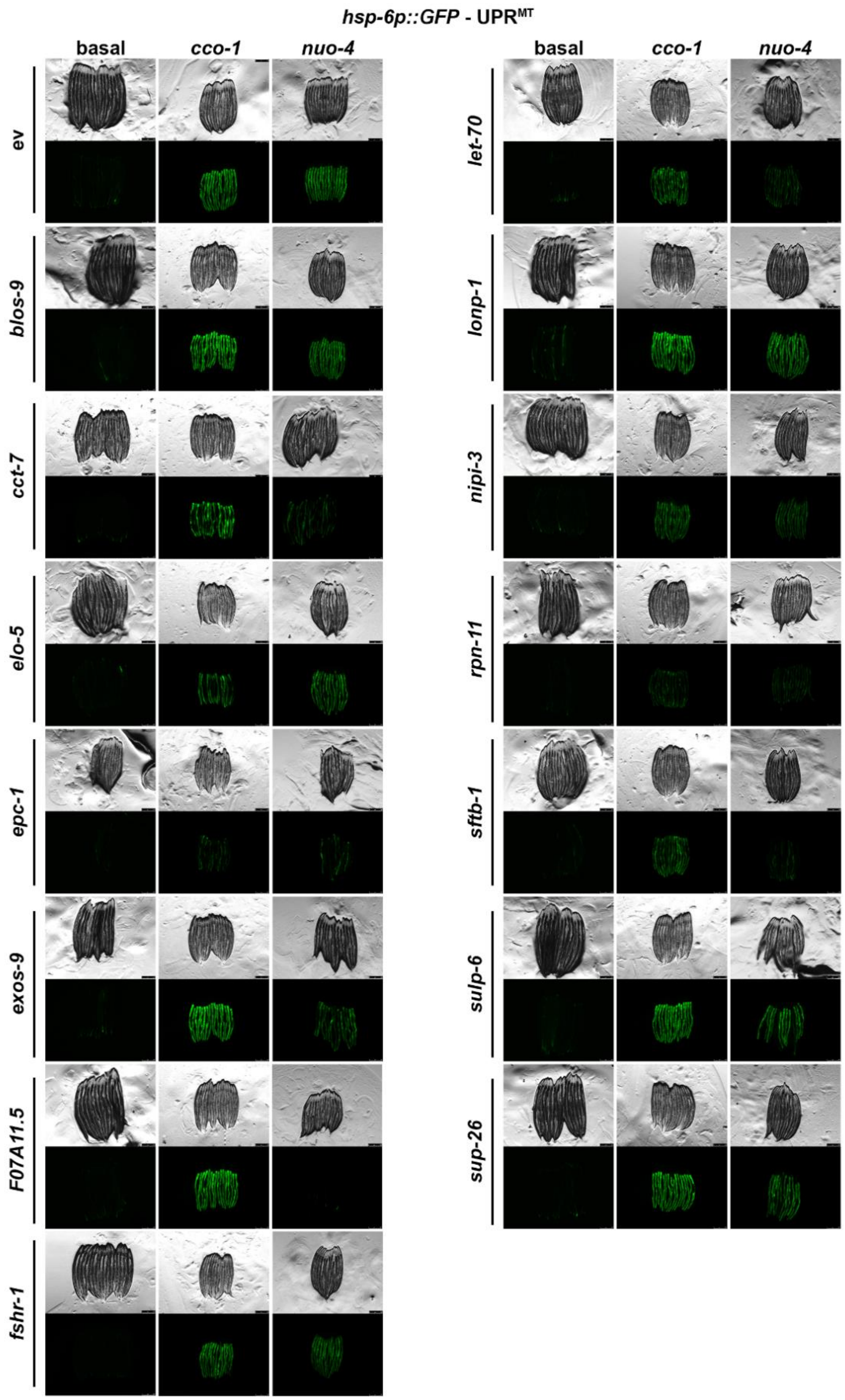

Supplemental File 5. UPR^{MT} Screen. *hsp-6p::GFP* animals were grown on RNAi indicated from hatch at 100% (basal) or mixed 50:50 with *cco*-1 or *nuo*-4 and imaged at day 1 of adulthood.

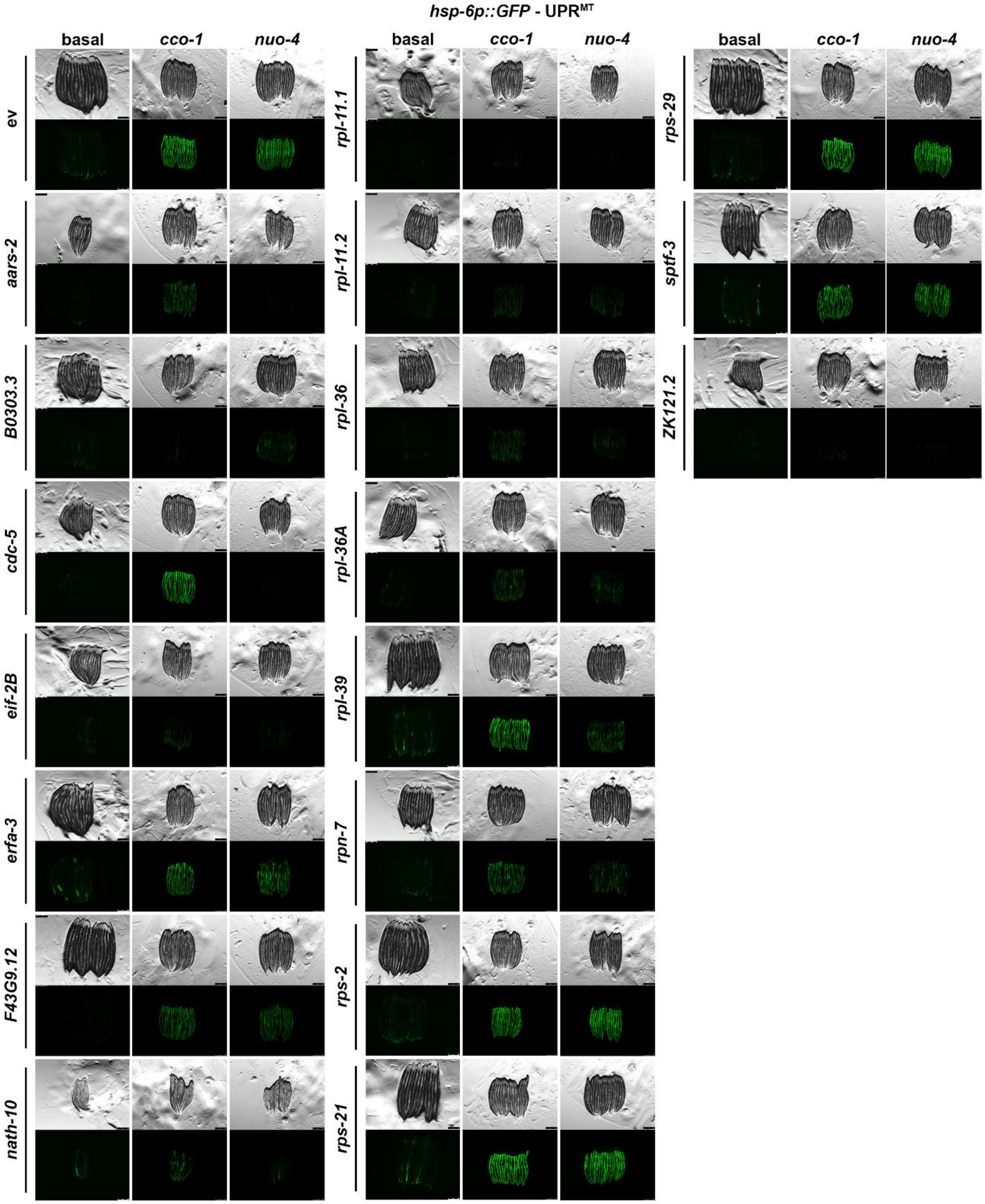

Supplemental File 6. UPR$^{\mathbf{MT}}$ **Screen.** *hsp-6p::GFP* animals were grown on RNAi indicated from hatch at 100% (basal) or mixed 50:50 with *cco*-1 or *nuo*-4 and imaged at day 1 of adulthood.

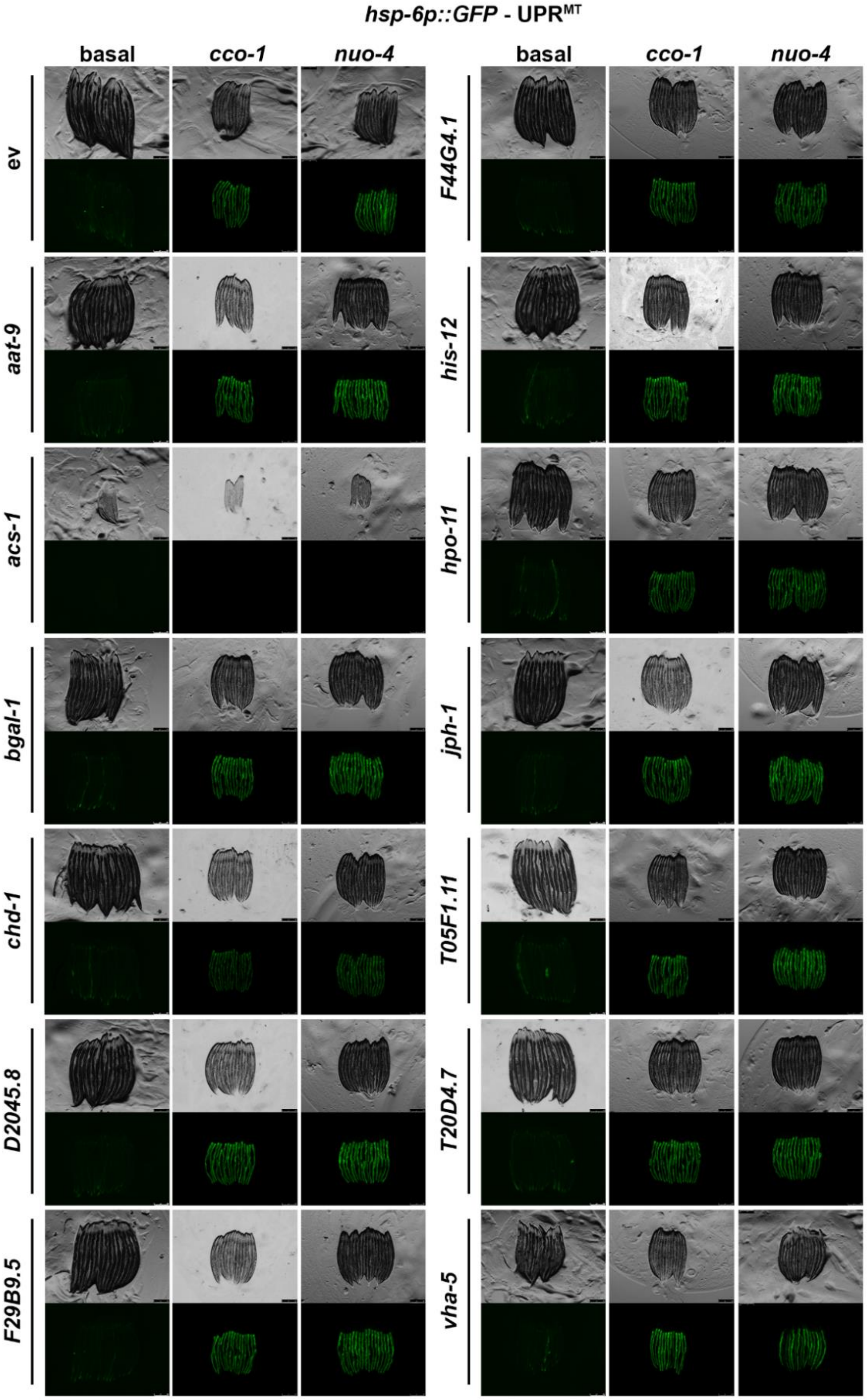

Supplemental File 7. UPR^{MT} Screen. *hsp-6p::GFP* animals were grown on RNAi indicated from hatch at 100% (basal) or mixed 50:50 with *cco*-1 or *nuo*-4 and imaged at day 1 of adulthood.

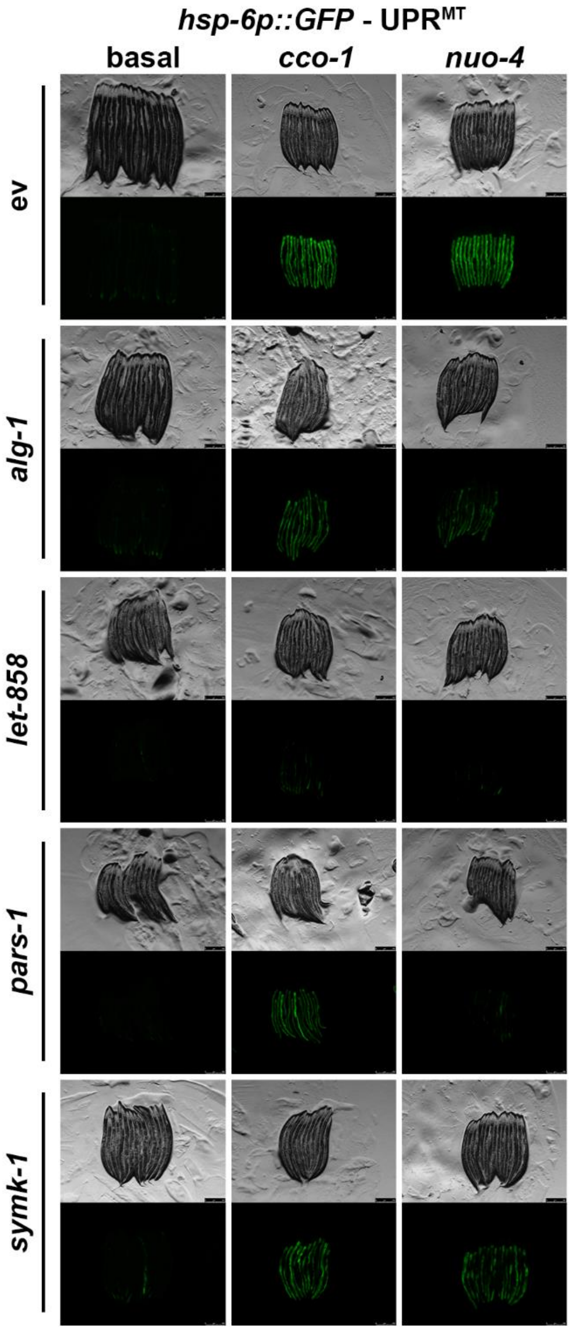

Supplemental File 8. UPR^{MT} Screen. *hsp-6p::GFP* animals were grown on RNAi indicated from hatch at 100% (basal) or mixed 50:50 with *cco*-1 or *nuo*-4 and imaged at day 1 of adulthood.

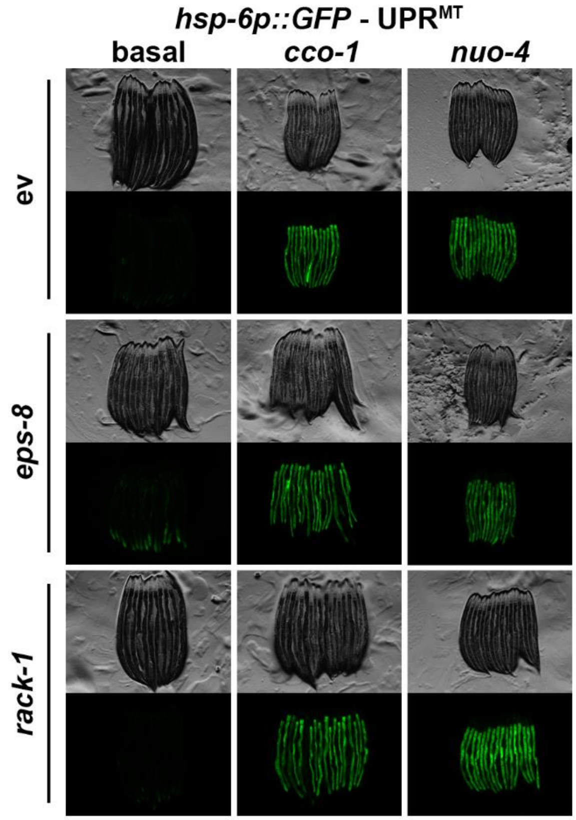

Supplemental File 9. UPR^{MT} Screen. *hsp-6p::GFP* animals were grown on RNAi indicated from hatch at 100% (basal) or mixed 50:50 with *cco*-1 or *nuo*-4 and imaged at day 1 of adulthood.

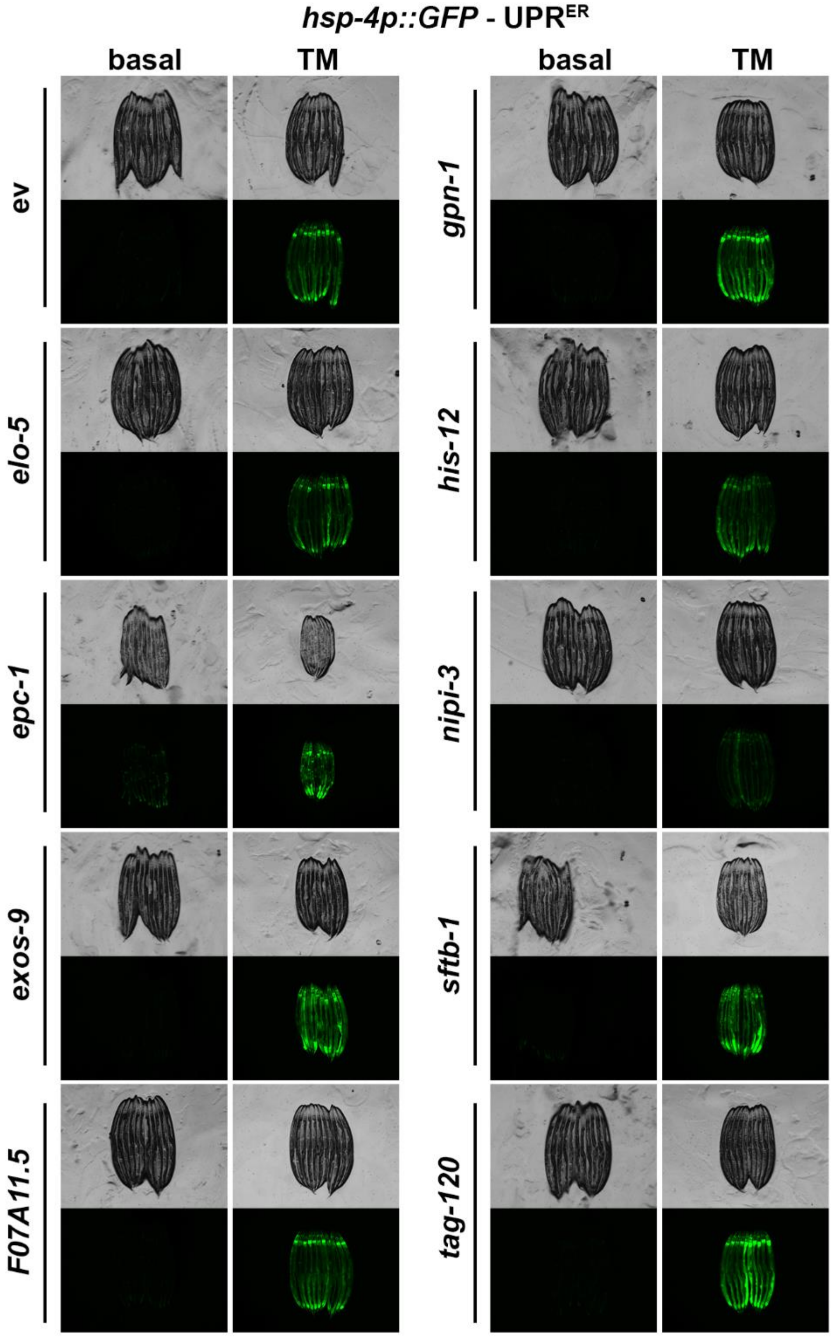

Supplemental File 10. UPR^{ER} Screen. *hsp-4p::GFP* animals were grown on RNAi indicated from hatch until L4. L4 animals were moved onto plates containing 25 ng/µL tunicamycin (TM) or left untreated (basal) and allowed to grow an additional 24 hours and imaged at day 1 of adulthood.

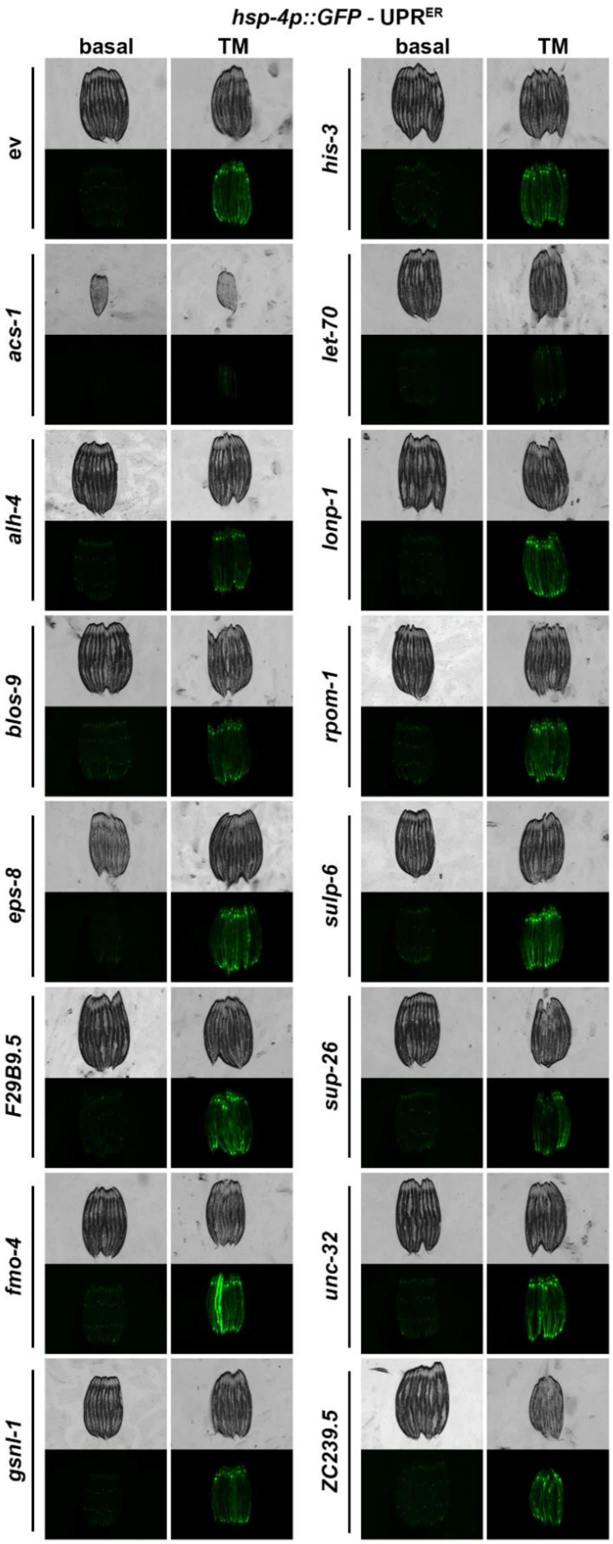

Supplemental File 11. UPR^{ER} Screen. *hsp-4p::GFP* animals were grown on RNAi indicated from hatch until L4. L4 animals were moved onto plates containing 25 ng/µL tunicamycin (TM) or left untreated (basal) and allowed to grow an additional 24 hours and imaged at day 1 of adulthood.

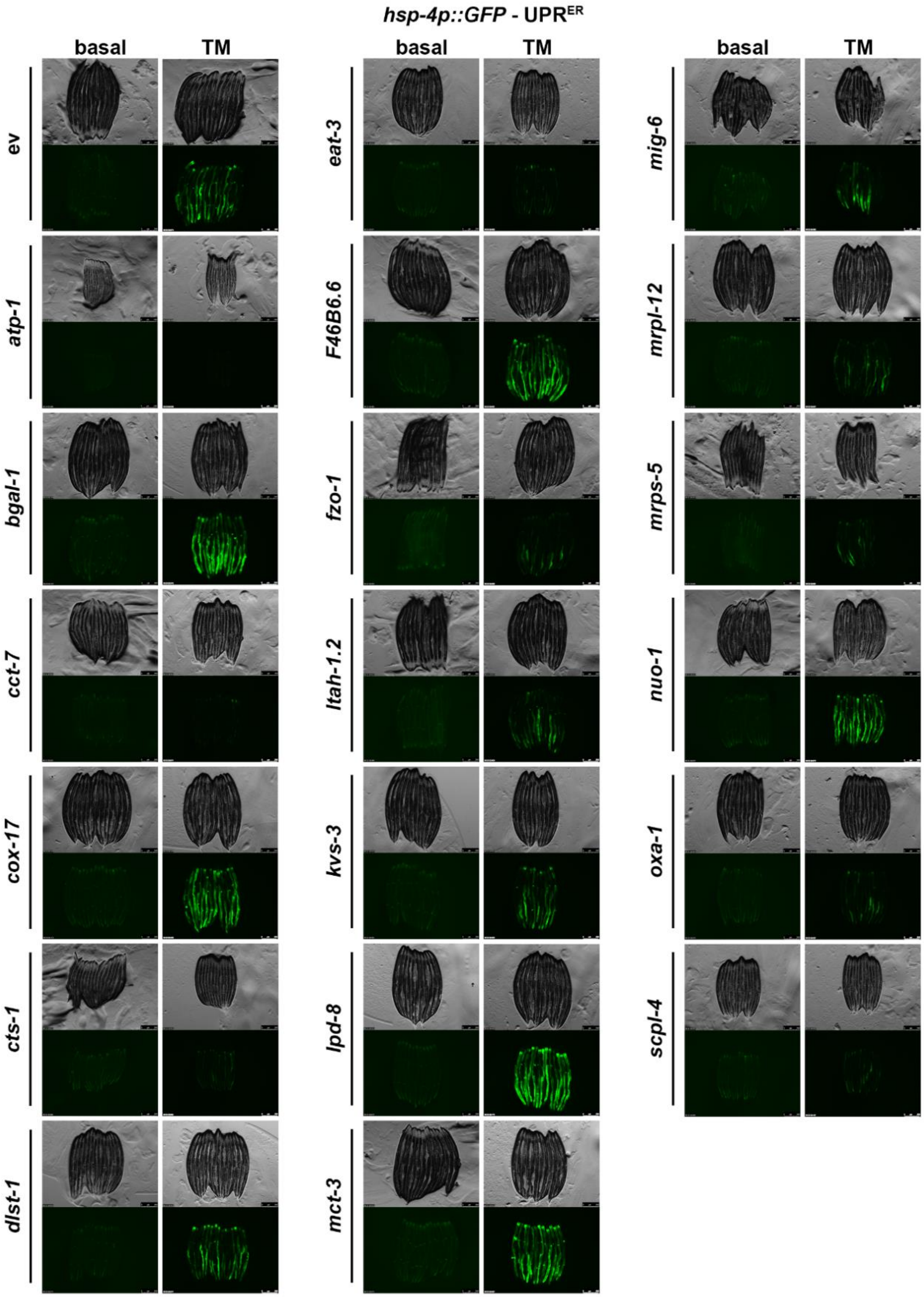

Supplemental File 12. UPR^{ER} Screen. *hsp-4p::GFP* animals were grown on RNAi indicated from hatch until L4. L4 animals were moved onto plates containing 25 ng/µL tunicamycin (TM) or left untreated (basal) and allowed to grow an additional 24 hours and imaged at day 1 of adulthood.

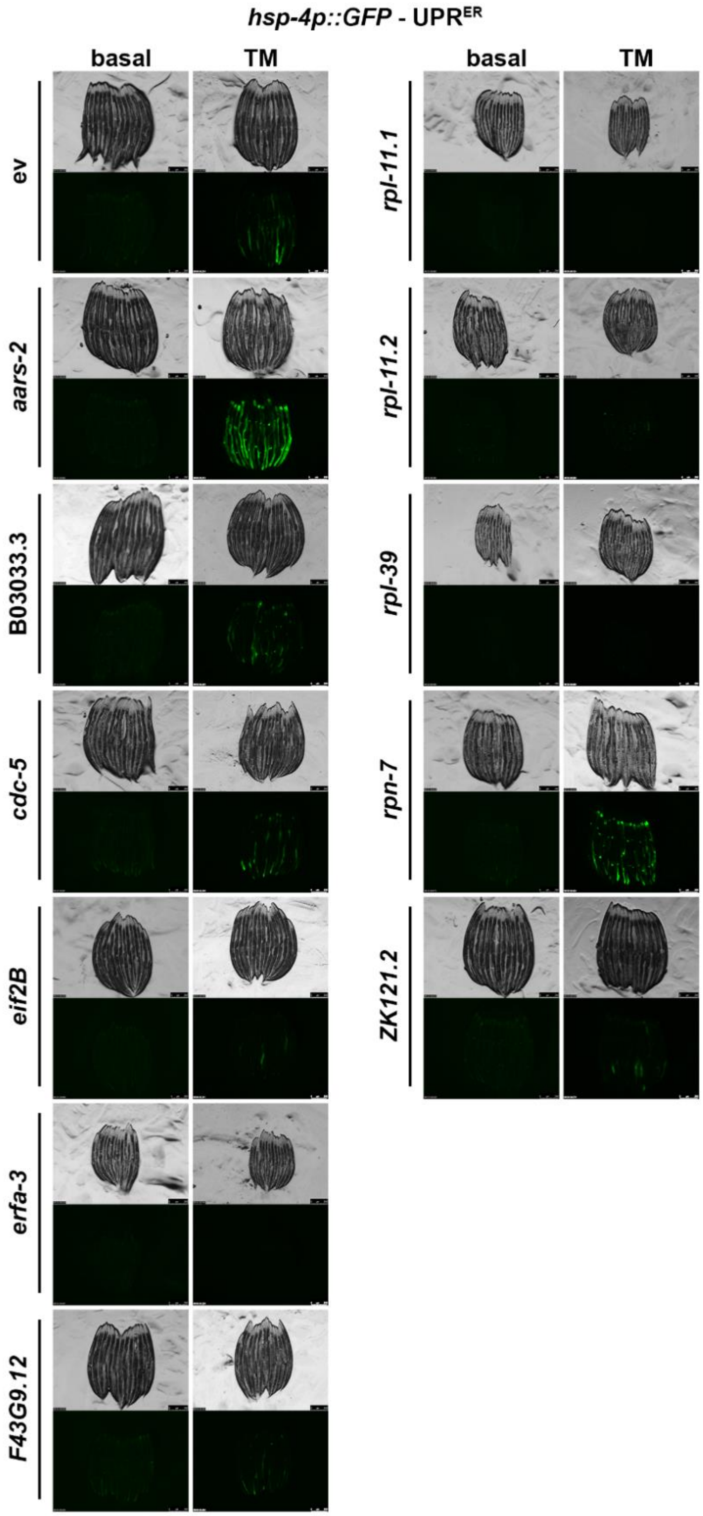

Supplemental File 13. UPR^{ER} Screen. *hsp-4p::GFP* animals were grown on RNAi indicated from hatch until L4. L4 animals were moved onto plates containing 25 ng/µL tunicamycin (TM) or left untreated (basal) and allowed to grow an additional 24 hours and imaged at day 1 of adulthood.

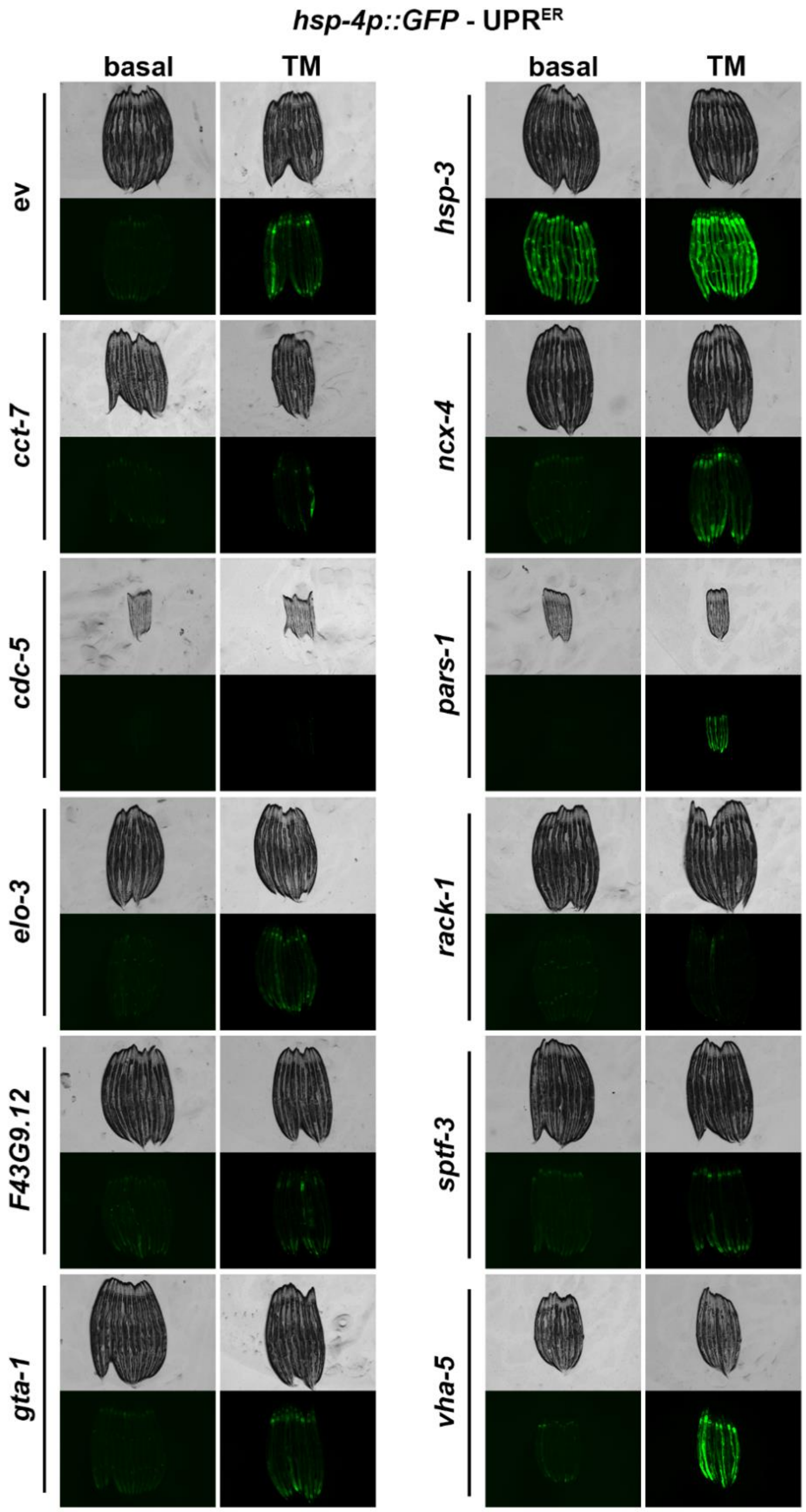

Supplemental File 14. UPR^{ER} Screen. *hsp-4p::GFP* animals were grown on RNAi indicated from hatch until L4. L4 animals were moved onto plates containing 25 ng/µL tunicamycin (TM) or left untreated (basal) and allowed to grow an additional 24 hours and imaged at day 1 of adulthood.

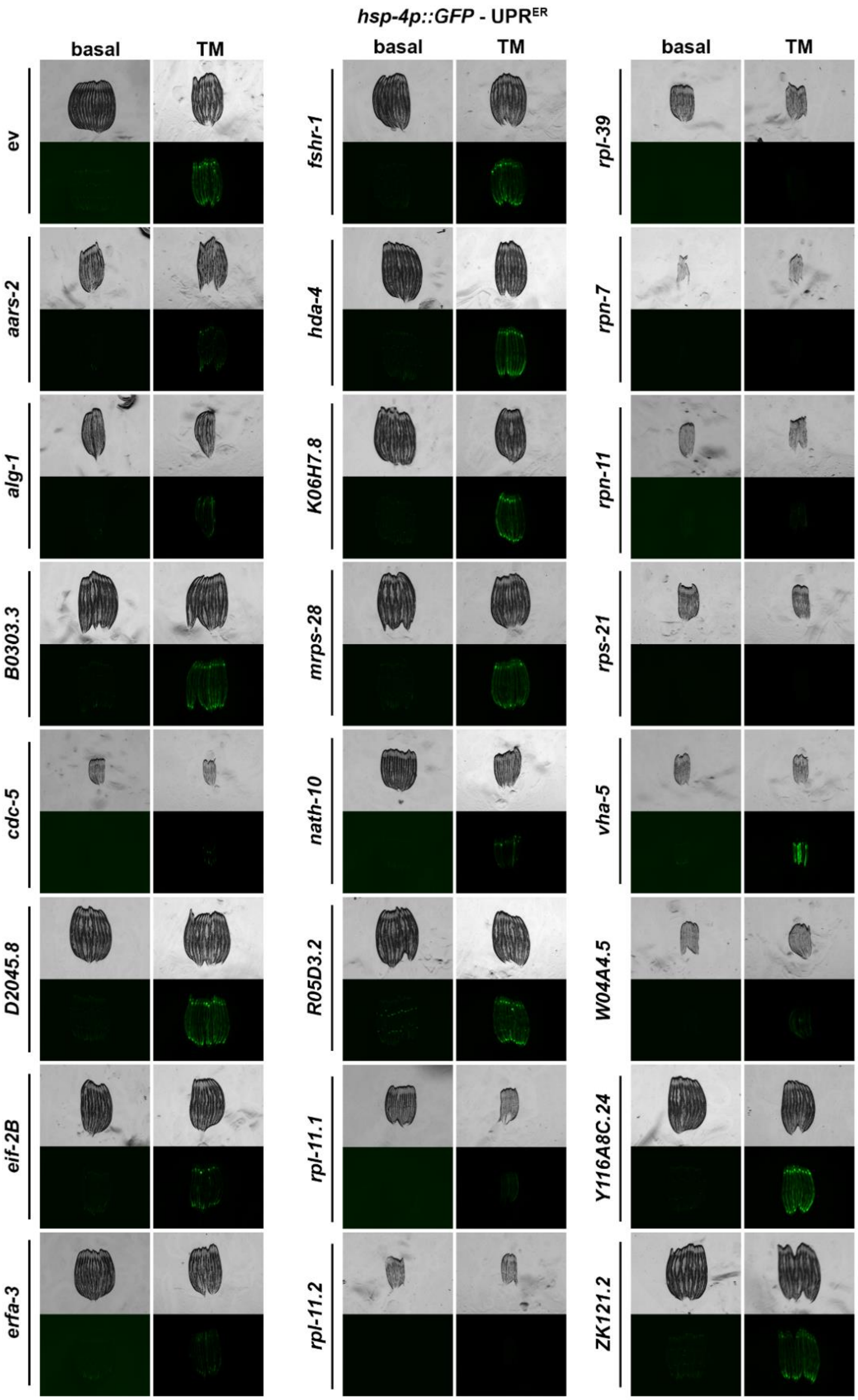

Supplemental File 15. UPR^{ER} Screen. *hsp-4p::GFP* animals were grown on RNAi indicated from hatch until L4. L4 animals were moved onto plates containing 25 ng/µL tunicamycin (TM) or left untreated (basal) and allowed to grow an additional 24 hours and imaged at day 1 of adulthood.

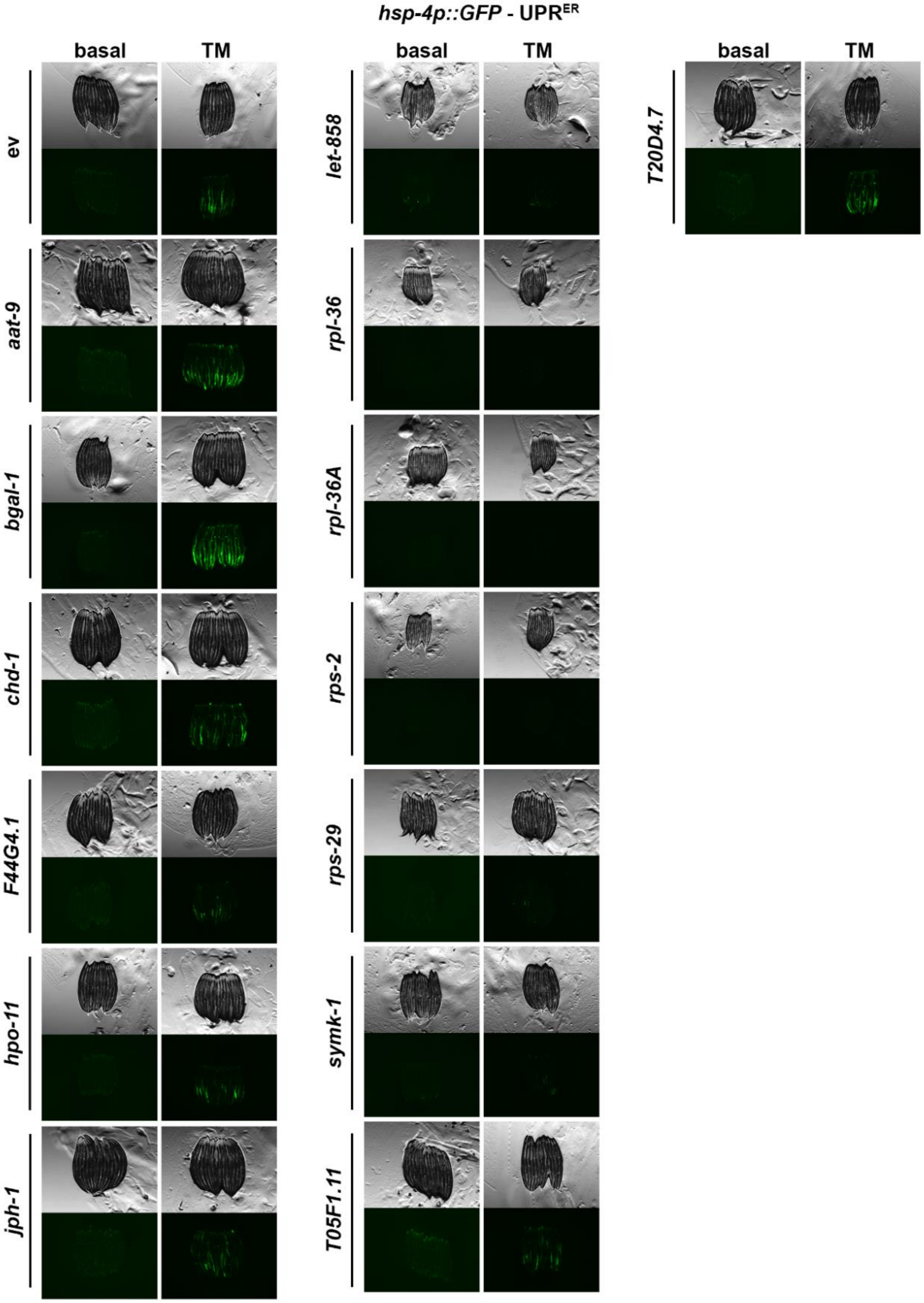

Supplemental File 16. UPR^{ER} Screen. *hsp-4p::GFP* animals were grown on RNAi indicated from hatch until L4. L4 animals were moved onto plates containing 25 ng/µL tunicamycin (TM) or left untreated (basal) and allowed to grow an additional 24 hours and imaged at day 1 of adulthood.

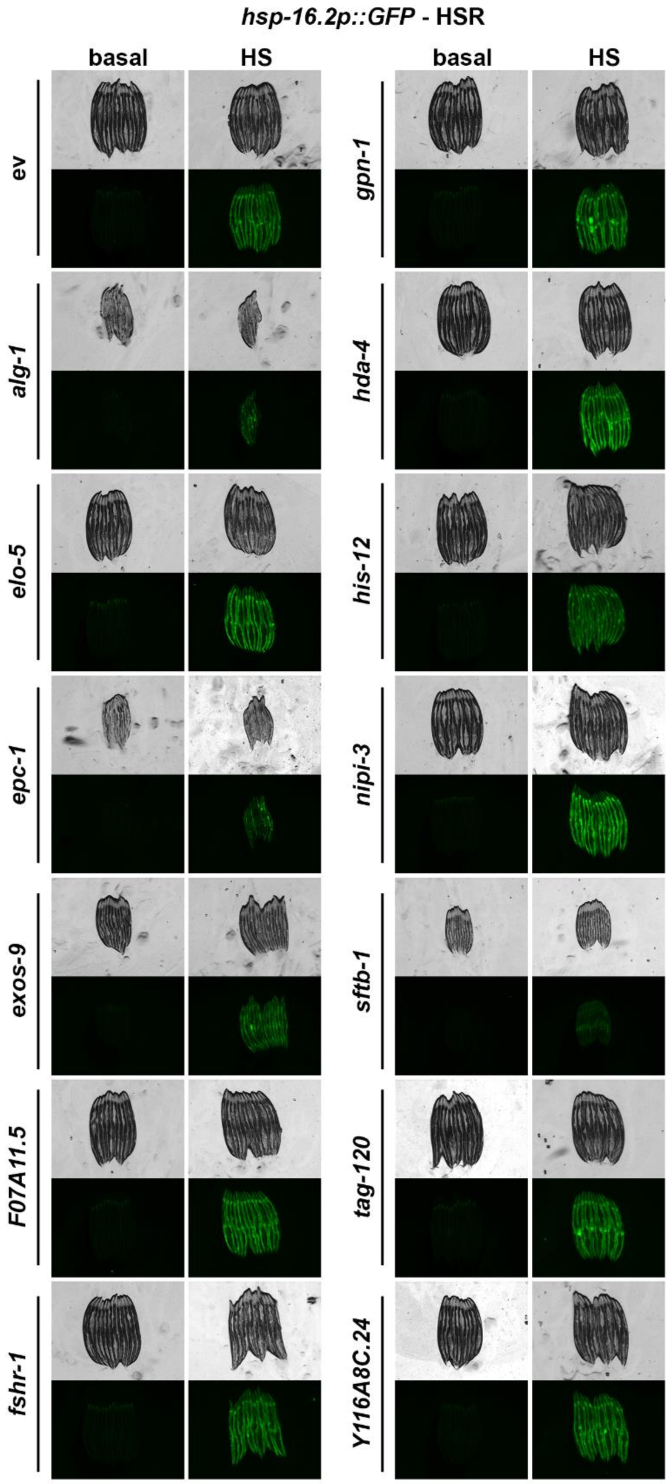

Supplemental File 17. HSR Screen. *hsp-16.2p::GFP* animals were grown on RNAi indicated from hatch until day 1. Day 1 adults were heat-shocked (HS) at 24 °C for two hours and recovered for two ours or left untreated (basal) prior to imaging.

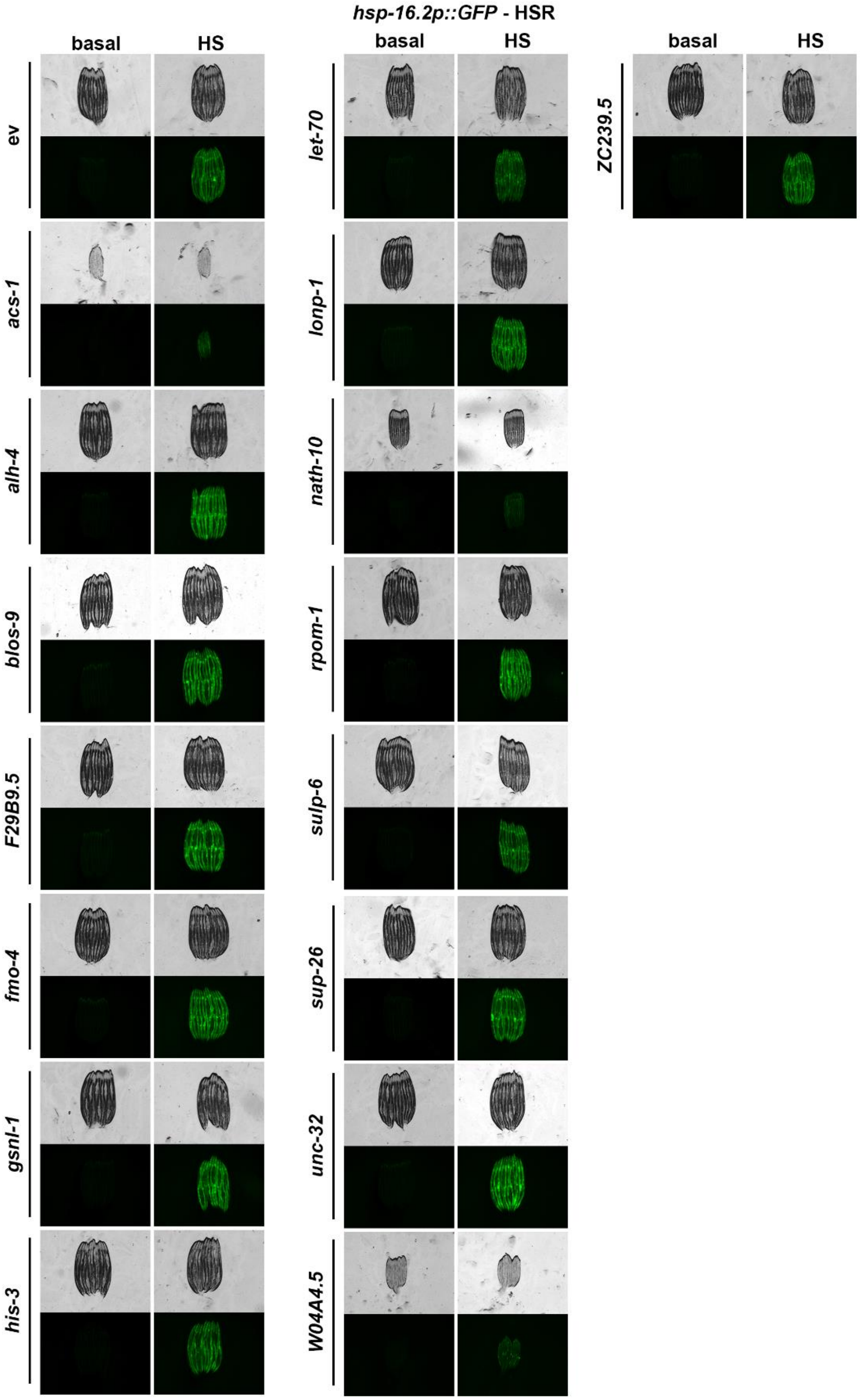

Supplemental File 18. HSR Screen. *hsp-16.2p::GFP* animals were grown on RNAi indicated from hatch until day 1. Day 1 adults were heat-shocked (HS) at 24 °C for two hours and recovered for two ours or left untreated (basal) prior to imaging.

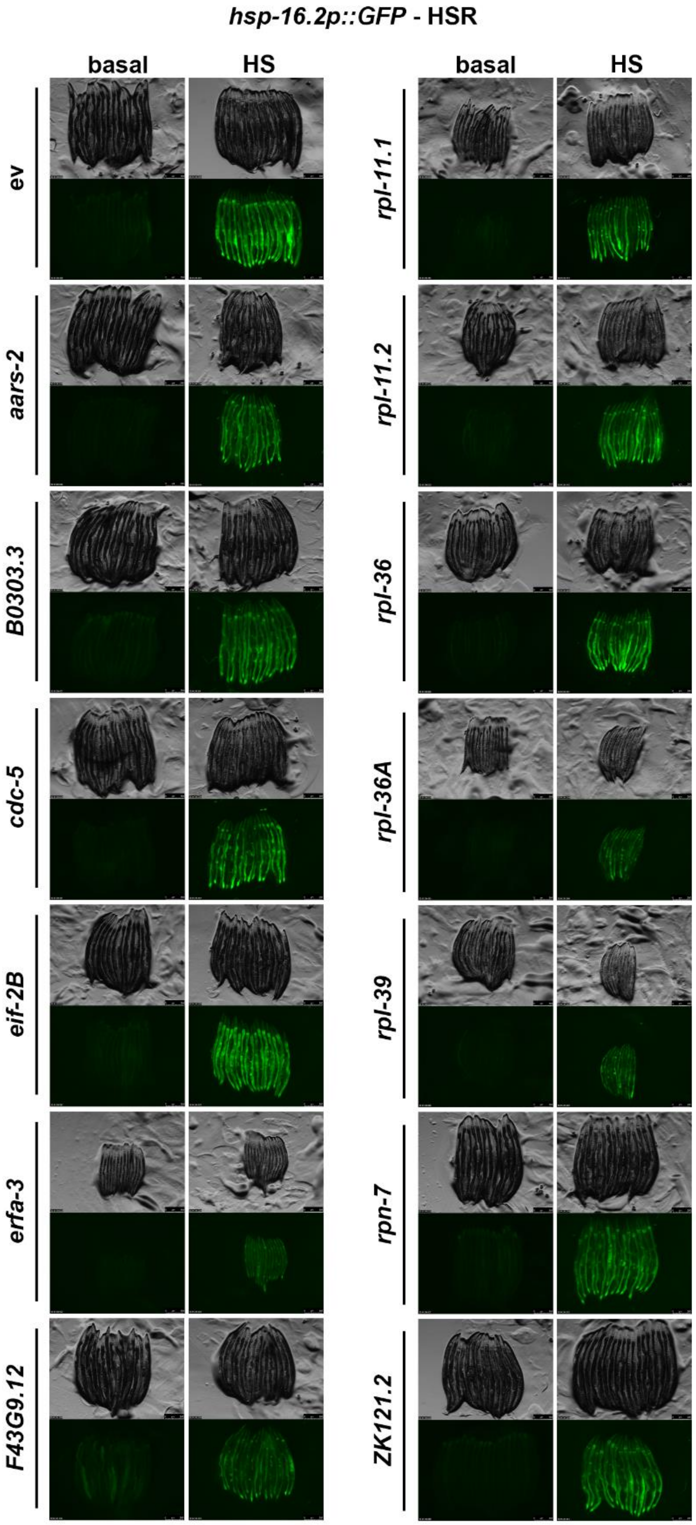

Supplemental File 19. HSR Screen. *hsp-16.2p::GFP* animals were grown on RNAi indicated from hatch until day 1. Day 1 adults were heat-shocked (HS) at 24 °C for two hours and recovered for two ours or left untreated (basal) prior to imaging.

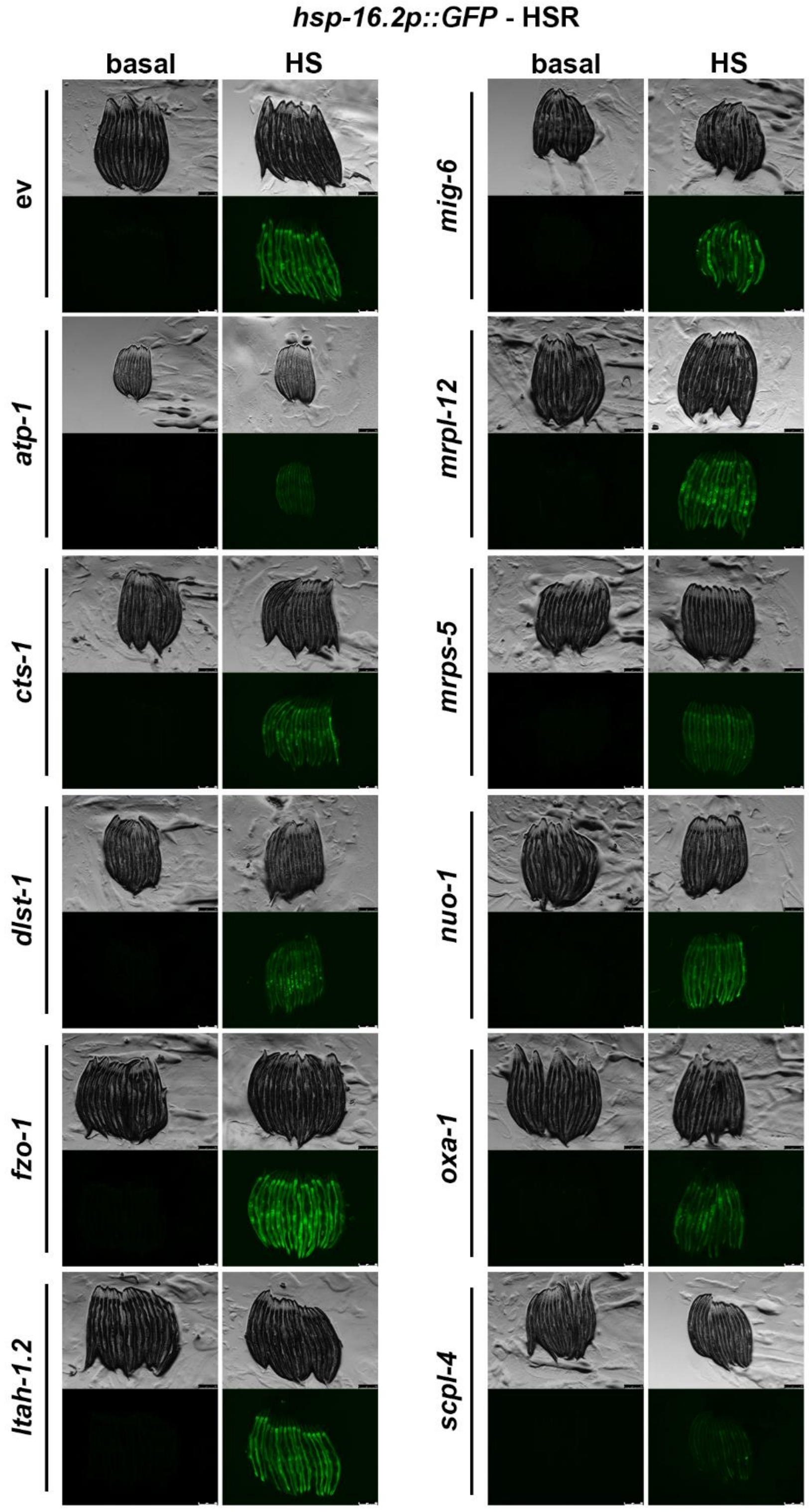

Supplemental File 20. HSR Screen. *hsp-16.2p::GFP* animals were grown on RNAi indicated from hatch until day 1. Day 1 adults were heat-shocked (HS) at 24 °C for two hours and recovered for two ours or left untreated (basal) prior to imaging.

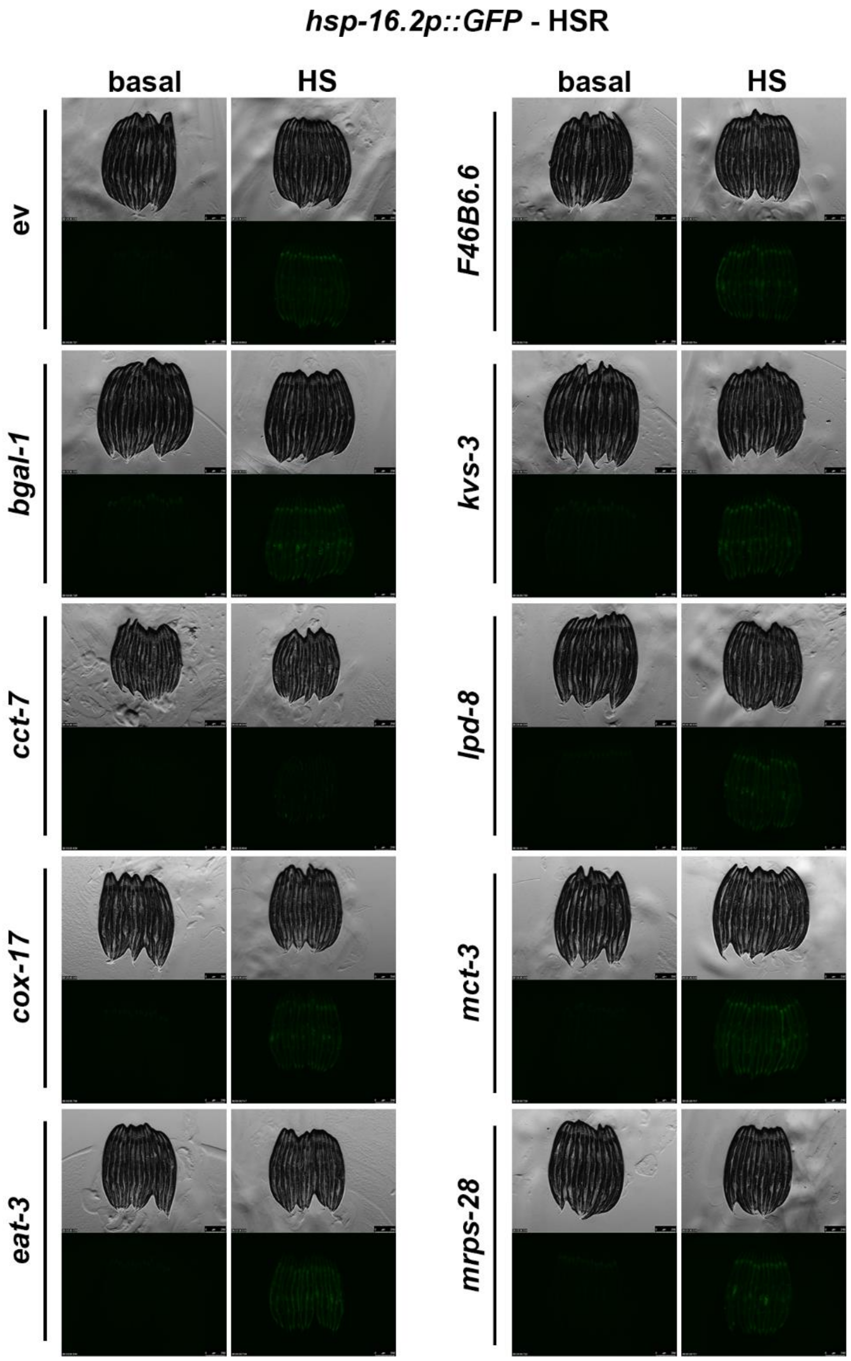

Supplemental File 21. HSR Screen. *hsp-16.2p::GFP* animals were grown on RNAi indicated from hatch until day 1. Day 1 adults were heat-shocked (HS) at 24 °C for two hours and recovered for two ours or left untreated (basal) prior to imaging.

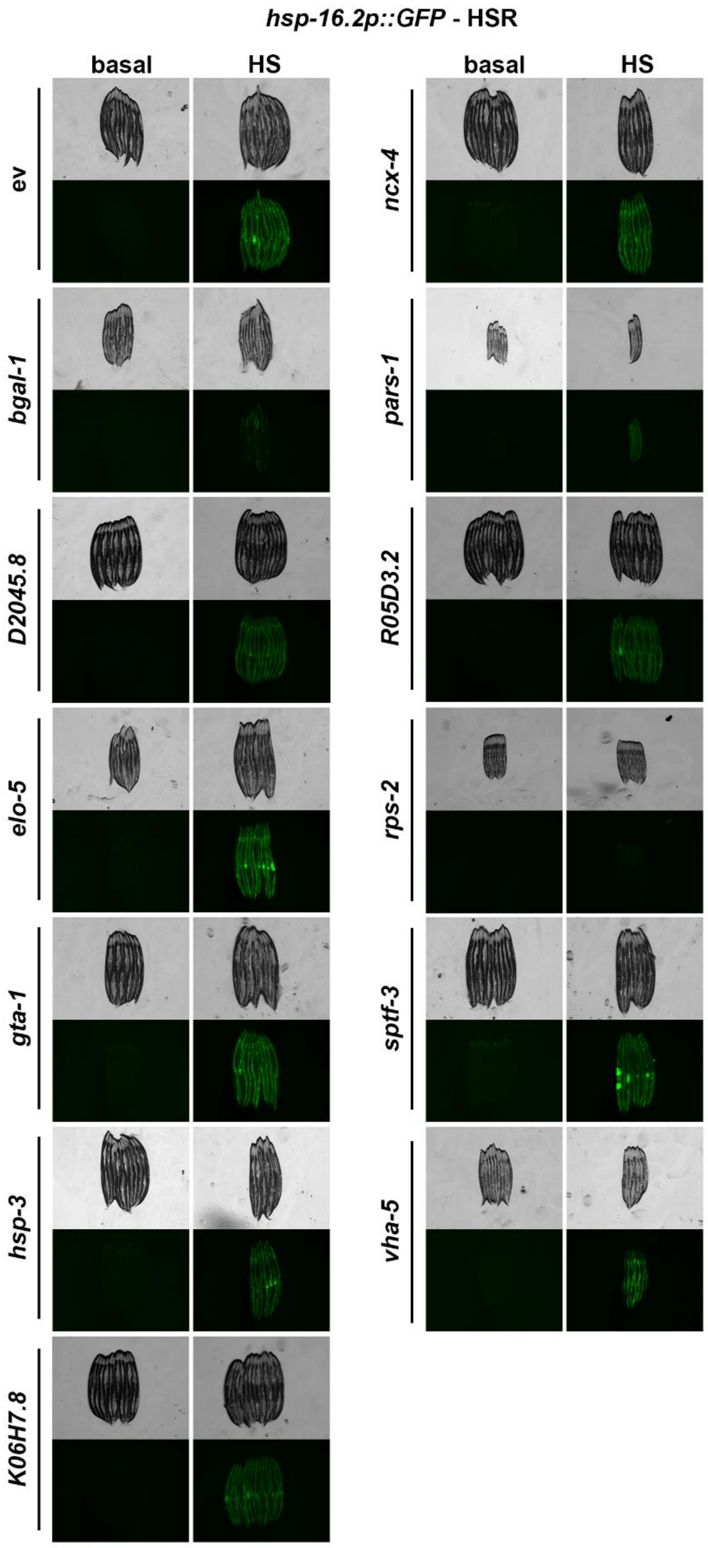

Supplemental File 22. HSR Screen. *hsp-16.2p::GFP* animals were grown on RNAi indicated from hatch until day 1. Day 1 adults were heat-shocked (HS) at 24 °C for two hours and recovered for two ours or left untreated (basal) prior to imaging.

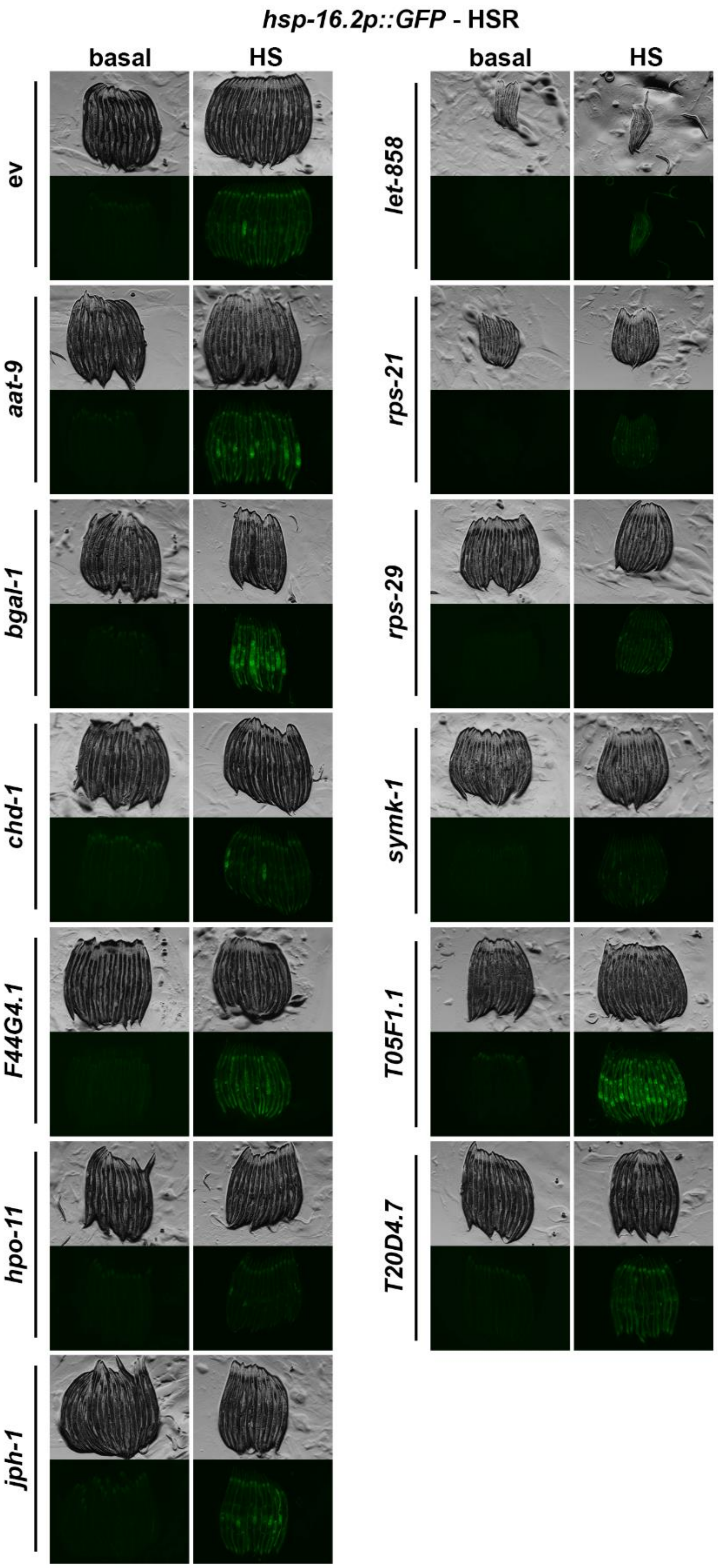

Supplemental File 23. HSR Screen. *hsp-16.2p::GFP* animals were grown on RNAi indicated from hatch until day 1. Day 1 adults were heat-shocked (HS) at 24 °C for two hours and recovered for two ours or left untreated (basal) prior to imaging.

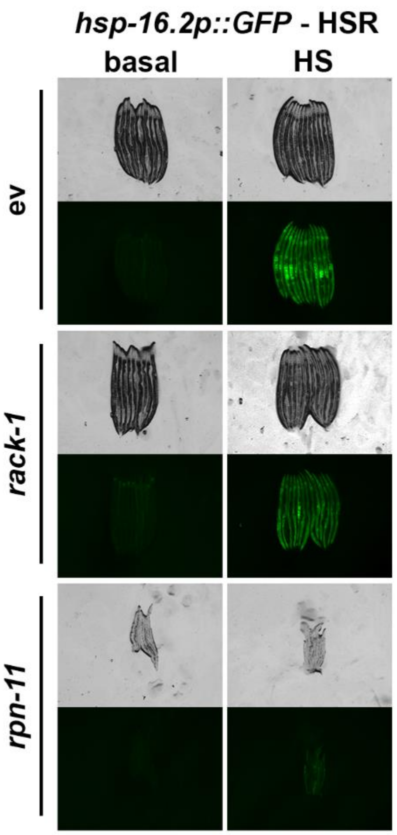

Supplemental File 24. HSR Screen. *hsp-16.2p::GFP* animals were grown on RNAi indicated from hatch until day 1. Day 1 adults were heat-shocked (HS) at 24 °C for two hours and recovered for two ours or left untreated (basal) prior to imaging.

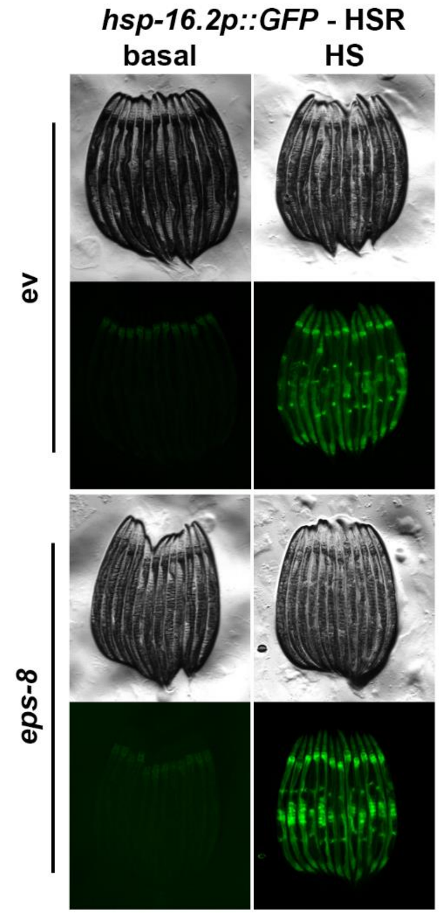

Supplemental File 25. HSR Screen. *hsp-16.2p::GFP* animals were grown on RNAi indicated from hatch until day 1. Day 1 adults were heat-shocked (HS) at 24 °C for two hours and recovered for two ours or left untreated (basal) prior to imaging.

REFERENCES AND NOTES

1. E. A. Moehle, K. Shen, A. Dillin, Mitochondrial proteostasis in the context of cellular and organismal health and aging. *J. Biol. Chem.* **294**, 5396–5407 (2019).

2. A. Dillin, A.-L. Hsu, N. Arantes-Oliveira, J. Lehrer-Graiwer, H. Hsin, A. G. Fraser, R. S. Kamath, J. Ahringer, C. Kenyon, Rates of behavior and aging specified by mitochondrial function during development. *Science* **298**, 2398–2401 (2002).

3. X. Liu, N. Jiang, B. Hughes, E. Bigras, E. Shoubridge, S. Hekimi, Evolutionary conservation of the clk-1-dependent mechanism of longevity: Loss of mclk1 increases cellular fitness and lifespan in mice. *Genes Dev.* **19**, 2424–2434 (2005).

4. E. Owusu-Ansah, W. Song, N. Perrimon, Muscle mitohormesis promotes longevity via systemic repression of insulin signaling. *Cell* **155**, 699–712 (2013).

5. C. Yee, W. Yang, S. Hekimi, The intrinsic apoptosis pathway mediates the pro-longevity response to mitochondrial ROS in *C. elegans*. *Cell* **157**, 897–909 (2014).

6. J. Durieux, S. Wolff, A. Dillin, The cell-non-autonomous nature of electron transport chain-mediated longevity. *Cell* **144**, 79–91 (2011).

7. A. M. Nargund, M. W. Pellegrino, C. J. Fiorese, B. M. Baker, C. M. Haynes, Mitochondrial import efficiency of ATFS-1 regulates mitochondrial UPR activation. *Science* **337**, 587–590 (2012).

8. C. Benedetti, C. M. Haynes, Y. Yang, H. P. Harding, D. Ron, Ubiquitin-like protein 5 positively regulates chaperone gene expression in the mitochondrial unfolded protein response. *Genetics* **174**, 229–239 (2006).

9. C. M. Haynes, K. Petrova, C. Benedetti, Y. Yang, D. Ron, ClpP mediates activation of a mitochondrial unfolded protein response in *C. elegans*. *Dev. Cell* **13**, 467–480 (2007).

10. Y. Tian, G. Garcia, Q. Bian, K. K. Steffen, L. Joe, S. Wolff, B. J. Meyer, A. Dillin, Mitochondrial stress induces chromatin reorganization to promote longevity and UPR(mt). *Cell* **165**, 1197–1208 (2016).

11. C. Merkwirth, V. Jovaisaite, J. Durieux, O. Matilainen, S. D. Jordan, P. M. Quiros, K. K. Steffen, E. G. Williams, L. Mouchiroud, S. U. Tronnes, V. Murillo, S. C. Wolff, R. J. Shaw, J. Auwerx, A. Dillin, Two conserved histone demethylases regulate mitochondrial stress-induced longevity. *Cell* **165**, 1209–1223 (2016).

12. C. J. Fiorese, A. M. Schulz, Y.-F. Lin, N. Rosin, M. W. Pellegrino, C. M. Haynes, The transcription factor ATF5 mediates a mammalian mitochondrial UPR. *Curr. Biol.* **26**, 2037–2043 (2016).

13. P. M. Quirós, M. A. Prado, N. Zamboni, D. D'Amico, R. W. Williams, D. Finley, S. P. Gygi, J. Auwerx, Multi-omics analysis identifies ATF4 as a key regulator of the mitochondrial stress response in mammals. *J. Cell Biol.* **216**, 2027–2045 (2017).

14. K. M. Tharp, R. Higuchi-Sanabria, G. Timblin, C. Garzon-Coral, B. Ford, C. Schneider, J. M. Muncie, C. Stashko, J. R. Daniele, P. A. Frankino, S. S. Manoli, H. Shao, J. Gestwicki, M. Hellerstein, D. K. Nomura, A. R. Dunn, K. Saijo, A. Dillin, V. M. Weaver, Adhesion-mediated mechanosignaling forces mitohormesis. bioRxiv 2020.03.06.979583 [Preprint]. 25 October 2020. https://doi.org/10.1101/2020.03.06.979583.

15. Y. M. Lages, J. M. Nascimento, G. A. Lemos, A. Galina, L. R. Castilho, S. K. Rehen, Low oxygen alters mitochondrial function and response to oxidative stress in human neural progenitor cells. *PeerJ.* **3**, e1486 (2015).

16. M. Khacho, R. Harris, R. S. Slack, Mitochondria as central regulators of neural stem cell fate and cognitive function. *Nat. Rev. Neurosci.* **20**, 34–48 (2019).

17. M. Khacho, A. Clark, D. S. Svoboda, J. Azzi, J. G. MacLaurin, C. Meghaizel, H. Sesaki, D. C. Lagace, M. Germain, M.-E. Harper, D. S. Park, R. S. Slack, Mitochondrial dynamics impacts stem cell identity and fate decisions by regulating a nuclear transcriptional program. *Cell Stem Cell* **19**, 232–247 (2016).

18. K. M. Berendzen, J. Durieux, L.-W. Shao, Y. Tian, H.-E. Kim, S. Wolff, Y. Liu, A. Dillin, Neuroendocrine coordination of mitochondrial stress signaling and proteostasis. *Cell* **166**, 1553–1563.e10 (2016).

19. Q. Zhang, X. Wu, P. Chen, L. Liu, N. Xin, Y. Tian, A. Dillin, The mitochondrial unfolded protein response is mediated cell-non-autonomously by retromer-dependent Wnt signaling. *Cell* **174**, 870–883.e17 (2018).

20. A. Naguib, G. Mathew, C. R. Reczek, K. Watrud, A. Ambrico, T. Herzka, I. C. Salas, M. F. Lee, N. El-Amine, W. Zheng, M. E. D. Francesco, J. R. Marszalek, D. J. Pappin, N. S. Chandel, L. C. Trotman, Mitochondrial complex I inhibitors expose a vulnerability for selective killing of Pten-null cells. *Cell Rep.* **23**, 58–67 (2018).

21. X.-Y. Sun, L. Wang, L. Cheng, N.-N. Li, Z.-J. Lu, J.-Y. Li, R. Peng, Genetic analysis of FGF20 in Chinese patients with Parkinson's disease. *Neurol. Sci.* **38**, 887–891 (2017).

22. S. J. Chia, E.-K. Tan, Y.-X. Chao, Historical perspective: Models of Parkinson's disease. *Int. J. Mol. Sci.* **21,** 2464 (2020).

23. K. Morita, Y. Hama, T. Izume, N. Tamura, T. Ueno, Y. Yamashita, Y. Sakamaki, K. Mimura, H. Morishita, W. Shihoya, O. Nureki, H. Mano, N. Mizushima, Genome-wide CRISPR screen identifies TMEM41B as a gene required for autophagosome formation. *J. Cell Biol.* **217**, 3817–3828 (2018).

24. W. Kim, R. S. Underwood, I. Greenwald, D. D. Shaye, OrthoList 2: A new comparative genomic analysis of human and *Caenorhabditis elegans* genes. *Genetics* **210**, 445–461 (2018).

25. R. Bar-Ziv, A. E. Frakes, R. Higuchi-Sanabria, T. Bolas, P. A. Frankino, H. K. Gildea, M. G. Metcalf, A. Dillin, Measurements of physiological stress responses in *C. elegans*. *J. Vis. Exp.*, e61001 (2020).

26. T. Yoneda, C. Benedetti, F. Urano, S. G. Clark, H. P. Harding, D. Ron, Compartment-specific perturbation of protein handling activates genes encoding mitochondrial chaperones. *J. Cell Sci.* **117**, 4055–4066 (2004).

27. D. E. Shore, C. E. Carr, G. Ruvkun, Induction of cytoprotective pathways is central to the extension of lifespan conferred by multiple longevity pathways. *PLOS Genet.* **8**, e1002792 (2012).

28. S. Haeussler, F. Köhler, M. Witting, M. F. Premm, S. G. Rolland, C. Fischer, L. Chauve, O. Casanueva, B. Conradt, Autophagy compensates for defects in mitochondrial dynamics. *PLOS Genet.* **16**, e1008638 (2020).

29. R. H. Houtkooper, L. Mouchiroud, D. Ryu, N. Moullan, E. Katsyuba, G. Knott, R. W. Williams, J. Auwerx, Mitonuclear protein imbalance as a conserved longevity mechanism. *Nature* **497**, 451–457 (2013).

30. S. Kim, D. Sieburth, Sphingosine kinase activates the mitochondrial unfolded protein response and is targeted to mitochondria by stress. *Cell Rep.* **24**, 2932–2945.e4 (2018).

31. A. B. Campos-Xavier, D. Martinet, J. Bateman, D. Belluoccio, L. Rowley, T. Y. Tan, A. Baxová, K.-H. Gustavson, Z. U. Borochowitz, A. M. Innes, S. Unger, J. S. Beckmann, L. Mittaz, D. Ballhausen, A. Superti-Furga, R. Savarirayan, L. Bonafé, Mutations in the heparan-sulfate proteoglycan glypican 6 (GPC6) impair endochondral ossification and cause recessive omodysplasia. *Am. J. Hum. Genet.* **84**, 760–770 (2009).

32. D. M. Tobin, J. C. Vary, J. P. Ray, G. S. Walsh, S. J. Dunstan, N. D. Bang, D. A. Hagge, S. Khadge, M.-C. King, T. R. Hawn, C. B. Moens, L. Ramakrishnan, The lta4h locus modulates susceptibility to mycobacterial infection in zebrafish and humans. *Cell* **140**, 717–730 (2010).

33. C. Couillault, P. Fourquet, M. Pophillat, J. J. Ewbank, A UPR-independent infection-specific role for a BiP/GRP78 protein in the control of antimicrobial peptide expression in *C. elegans* epidermis. *Virulence* **3**, 299–308 (2012).

34. Y. Chen, Z. Yang, M. Meng, Y. Zhao, N. Dong, H. Yan, L. Liu, M. Ding, H. B. Peng, F. Shao, Cullin mediates degradation of RhoA through evolutionarily conserved BTB adaptors to control actin cytoskeleton structure and cell movement. *Mol. Cell* **35**, 841–855 (2009).

35. M. Bouskila, N. Esoof, L. Gay, E. H. Fang, M. Deak, M. J. Begley, L. C. Cantley, A. Prescott, K. G. Storey, D. R. Alessi, TTBK2 kinase substrate specificity and the impact of spinocerebellar-ataxia-causing mutations on expression, activity, localization and development. *Biochem. J.* **437**, 157–167 (2011).

36. M. Calfon, H. Zeng, F. Urano, J. H. Till, S. R. Hubbard, H. P. Harding, S. G. Clark, D. Ron, IRE1 couples endoplasmic reticulum load to secretory capacity by processing the XBP-1 mRNA. *Nature* **415**, 92–96 (2002).

37. A. Heifetz, R. W. Keenan, A. D. Elbein, Mechanism of action of tunicamycin on the UDP-GlcNAc:dolichyl-phosphate Glc-NAc-1-phosphate transferase. *Biochemistry* **18**, 2186–2192 (1979).

38. C. D. Link, J. R. Cypser, C. J. Johnson, T. E. Johnson, Direct observation of stress response in *Caenorhabditis elegans* using a reporter transgene. *Cell Stress Chaperones* **4**, 235–242 (1999).

39. H.-E. Kim, A. R. Grant, M. S. Simic, R. A. Kohnz, D. K. Nomura, J. Durieux, C. E. Riera, M. Sanchez, E. Kapernick, S. Wolff, A. Dillin, Lipid biosynthesis coordinates a mitochondrial-to-cytosolic stress response. *Cell* **166**, 1539–1552.e16 (2016).

40. J. Labbadia, R. M. Brielmann, M. F. Neto, Y.-F. Lin, C. M. Haynes, R. I. Morimoto, Mitochondrial stress restores the heat shock response and prevents proteostasis collapse during aging. *Cell Rep.* **21**, 1481–1494 (2017).

41. S. Niwa, L. Tao, S. Y. Lu, G. M. Liew, W. Feng, M. V. Nachury, K. Shen, BORC regulates the axonal transport of synaptic vesicle precursors by activating ARL-8. *Curr. Biol.* **27**, 2569–2578.e4 (2017).

42. M. Kniazeva, Q. T. Crawford, M. Seiber, C.-Y. Wang, M. Han, Monomethyl branched-chain fatty acids play an essential role in *Caenorhabditis elegans* development. *PLoS Biol.* **2**, E257 (2004).

43. Y. Doyon, W. Selleck, W. S. Lane, S. Tan, J. Côté, Structural and functional conservation of the NuA4 histone acetyltransferase complex from yeast to humans. *Mol. Cell. Biol.* **24**, 1884–1896 (2004).

44. D. Mukherjee, M. Gao, J. P. O’Connor, R. Raijmakers, G. Pruijn, C. S. Lutz, J. Wilusz, The mammalian exosome mediates the efficient degradation of mRNAs that contain AU-rich elements. *EMBO J.* **21**, 165–174 (2002).

45. R. Das, Z. Zhou, R. Reed, Functional association of U2 snRNP with the ATP-independent spliceosomal complex E. *Mol. Cell* **5**, 779–787 (2000).

46. N. Ohoka, S. Yoshii, T. Hattori, K. Onozaki, H. Hayashi, TRB3, a novel ER stress-inducible gene, is induced via ATF4-CHOP pathway and is involved in cell death. *EMBO J.* **24**, 1243–1255 (2005).

47. A. M. Nargund, C. J. Fiorese, M. W. Pellegrino, P. Deng, C. M. Haynes, Mitochondrial and nuclear accumulation of the transcription factor ATFS-1 promotes OXPHOS recovery during the UPR(mt). *Mol. Cell* **58**, 123–133 (2015).

48. R. Higuchi-Sanabria, J. Durieux, N. Kelet, S. Homentcovschi, M. de Los Rios Rogers, S. Monshietehadi, G. Garcia, S. Dallarda, J. R. Daniele, V. Ramachandran, A. Sahay, S. U. Tronnes, L. Joe, A. Dillin, Divergent Nodes of Non-autonomous UPR^{ER} signaling through serotonergic and dopaminergic neurons. *Cell Rep.* **33**, 108489 (2020).

49. L. Kussmaul, J. Hirst, The mechanism of superoxide production by NADH:ubiquinone oxidoreductase (complex I) from bovine heart mitochondria. *Proc. Natl. Acad. Sci. U.S.A.* **103**, 7607–7612 (2006).

50. M. D. Brand, The sites and topology of mitochondrial superoxide production. *Exp. Gerontol.* **45**, 466–472 (2010).

51. M. P. Murphy, How mitochondria produce reactive oxygen species. *Biochem. J.* **417**, 1–13 (2009).

52. C. D. Link, C. J. Johnson, Reporter transgenes for study of oxidant stress in *Caenorhabditis elegans*. *Methods Enzymol.* **353**, 497–505 (2002).

53. S. Koyuncu, R. Loureiro, H. J. Lee, P. Wagle, M. Krueger, D. Vilchez, Rewiring of the ubiquitinated proteome determines ageing in *C. elegans*. *Nature* **596**, 285–290 (2021).

54. G. Scita, P. Tenca, L. B. Areces, A. Tocchetti, E. Frittoli, G. Giardina, I. Ponzanelli, P. Sini, M. Innocenti, P. P. Di Fiore, An effector region in Eps8 is responsible for the activation of the Rac-specific GEF activity of Sos-1 and for the proper localization of the Rac-based actin–polymerizing machine. *J. Cell Biol.* **154**, 1031–1044 (2001).

55. N. A. Mack, H. J. Whalley, S. Castillo-Lluva, A. Malliri, The diverse roles of Rac signaling in tumorigenesis. *Cell Cycle* **10**, 1571–1581 (2011).

56. A. Palamidessi, E. Frittoli, N. Ducano, N. Offenhauser, S. Sigismund, H. Kajiho, D. Parazzoli, A. Oldani, M. Gobbi, G. Serini, P. P. Di Fiore, G. Scita, L. Lanzetti, The GTPase-activating protein RN-tre controls focal adhesion turnover and cell migration. *Curr. Biol.* **23**, 2355–2364 (2013).

57. N. R. Paul, J. R. Thomas, H. Maldonado, K. I. Wolanska, E. J. Koper, J. D. Humphries, A. Byron, A. George, N. Allen, I. A. Prior, C. H. Streuli, M. J. Humphries, M. R. Morgan, Eps8 is a convergence point integrating EGFR and integrin trafficking and crosstalk. bioRxiv 405043 [Preprint]. 4 September 2018. https://doi.org/10.1101/405043.

58. R. Higuchi-Sanabria, J. W. Paul Rd, J. Durieux, C. Benitez, P. A. Frankino, S. U. Tronnes, G. Garcia, J. R. Daniele, S. Monshietehadi, A. Dillin, Spatial regulation of the actin cytoskeleton by HSF-1 during aging. *Mol. Biol. Cell* **29**, 2522–2527 (2018).

59. T. R. Arnold, R. E. Stephenson, A. L. Miller, Rho GTPases and actomyosin: Partners in regulating epithelial cell-cell junction structure and function. *Exp. Cell Res.* **358**, 20–30 (2017).

60. J. A. Thomson, J. Itskovitz-Eldor, S. S. Shapiro, M. A. Waknitz, J. J. Swiergiel, V. S. Marshall, J. M. Jones, Embryonic stem cell lines derived from human blastocysts. *Science* **282**, 1145–1147 (1998).

61. S. M. Chambers, C. A. Fasano, E. P. Papapetrou, M. Tomishima, M. Sadelain, L. Studer, Highly efficient neural conversion of human ES and iPS cells by dual inhibition of SMAD signaling. *Nat. Biotechnol.* **27**, 275–280 (2009).

62. J. G. Doench, N. Fusi, M. Sullender, M. Hegde, E. W. Vaimberg, K. F. Donovan, I. Smith, Z. Tothova, C. Wilen, R. Orchard, H. W. Virgin, J. Listgarten, D. E. Root, Optimized sgRNA design to maximize activity and minimize off-target effects of CRISPR-Cas9. *Nat. Biotechnol.* **34**, 184–191 (2016).

63. R. T. Schinzel, R. Higuchi-Sanabria, O. Shalem, E. A. Moehle, B. M. Webster, L. Joe, R. Bar-Ziv, P. A. Frankino, J. Durieux, C. Pender, N. Kelet, S. S. Kumar, N. Savalia, H. Chi, M. Simic, N.-T.

Nguyen, A. Dillin, The hyaluronidase, TMEM2, promotes ER homeostasis and longevity independent of the UPRER. *Cell* **179**, 1306–1318.e18 (2019).

64. R. Staden, Computer methods to locate signals in nucleic acid sequences. *Nucleic Acids Res.* **12**, 505–519 (1984).

65. S. Neph, M. S. Kuehn, A. P. Reynolds, E. Haugen, R. E. Thurman, A. K. Johnson, E. Rynes, M. T. Maurano, J. Vierstra, S. Thomas, R. Sandstrom, R. Humbert, J. A. Stamatoyannopoulos, BEDOPS: High-performance genomic feature operations. *Bioinformatics* **28**, 1919–1920 (2012).

66. M. T. Maurano, E. Haugen, R. Sandstrom, J. Vierstra, A. Shafer, R. Kaul, J. A. Stamatoyannopoulos, Large-scale identification of sequence variants influencing human transcription factor occupancy in vivo. *Nat. Genet.* **47**, 1393–1401 (2015).

67. S. Urlinger, U. Baron, M. Thellmann, M. T. Hasan, H. Bujard, W. Hillen, Exploring the sequence space for tetracycline-dependent transcriptional activators: Novel mutations yield expanded range and sensitivity. *Proc. Natl. Acad Sci. U.S.A.* **97**, 7963–7968 (2000).

68. O. Y. Wouters, D. T. A. Ploeger, S. M. van Putten, R. A. Bank, 3,4-Dihydroxy-l-phenylalanine as a novel covalent linker of extracellular matrix proteins to polyacrylamide hydrogels with a tunable stiffness. *Tissue Eng. Part C Methods* **22**, 91–101 (2016).

69. S. L. Rea, N. Ventura, T. E. Johnson, Relationship between mitochondrial electron transport chain dysfunction, development, and life extension in *Caenorhabditis elegans*. *PLOS Biol.* **5,** e259 (2007).

70. Y. Zhang, A. Lanjuin, S. R. Chowdhury, M. Mistry, C. G. Silva-García, H. J. Weir, C.-L. Lee, C. C. Escoubas, E. Tabakovic, W. B. Mair, Neuronal TORC1 modulates longevity via AMPK and cell nonautonomous regulation of mitochondrial dynamics in *C. elegans*. *eLife* **8**, e49158 (2019).

71. X. Shen, R. E. Ellis, K. Sakaki, R. J. Kaufman, Genetic interactions due to constitutive and inducible gene regulation mediated by the unfolded protein response in *C. elegans*. *PLOS Genet.* **1**, e37 (2005).
